# Dye Extraction by Functionalized Magnetic Nanoparticles with Surfactants and Cyclodextrins through Specific and Host–Guest Interactions

**DOI:** 10.1021/acs.langmuir.4c05340

**Published:** 2025-03-22

**Authors:** Manoj Kumar Goshisht, Rajpreet Kaur, Mandeep Singh Bakshi

**Affiliations:** Department of Chemistry, Natural and Applied Sciences, University of Wisconsin - Green Bay, 2420 Nicolet Drive, Green Bay, Wisconsin 54311-7001, United States

## Abstract

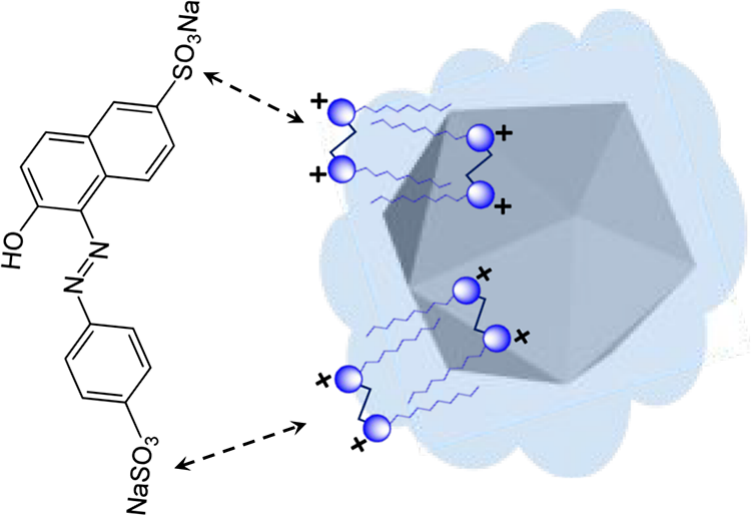

Functionalized magnetic nanoparticles (NPs) were synthesized by using different surfactants and cyclodextrins (CDs) for the extraction of toxic dyes from the aqueous bulk at room temperature to exploit the surfactant/CD–dye interactions operating at the solid–liquid interface. The extraction of dyes was monitored by UV–visible studies and quantitatively estimated. Both hexamethylene bis(hexadecyl dimethylammonium bromide) (16–6–16) and tris(2-(N-dodecyl *N*,*N*-dimethylammonio)ethylamine) tribromide (TriCAT) functionalized magnetic NPs proved to be excellent extractors, whereas extraction was highly facilitated at high or low pH when CD-functionalized magnetic NPs were used. Self-association among the dye molecules impeded the extraction. A combination of both polar as well as nonpolar along with dye–dye interactions participated in the extraction. Quantification was carried out by performing surface analysis of dye-loaded magnetic NPs. FTIR and XPS identified the functionalities participating in the extraction, whereas TEM, FESEM, and their EDS analyses determined the elemental composition. The amounts of N and S acted as fingerprinting elements for the adsorption of dyes at the solid–liquid interface, whereas overall amounts of C and O qualitatively differentiated among the relative amounts of dyes extracted. All results concluded that the extraction was much facilitated for those dyes which demonstrated a low degree of self-association in aqueous bulk and a high degree of solid–liquid interfacial adsorption.

## Introduction

**Scheme 1:**
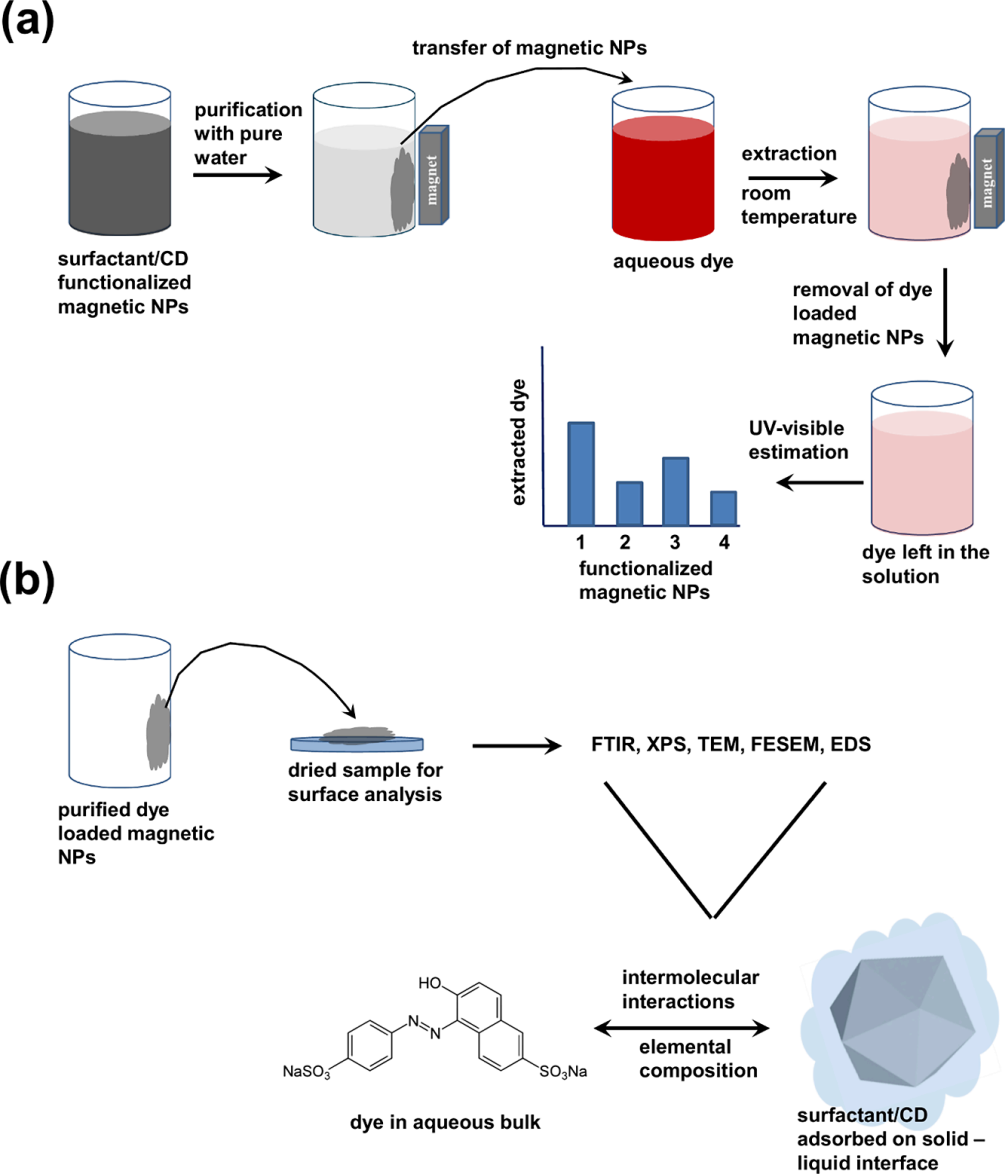
(a) Flow Diagram Showing the Use of Surfactant/CD-Functionalized Magnetic NPs in the Extraction Process. The Magnetic NPs are First Purified and Collected with the External Magnet; They Are Placed in a Series of Aqueous Solutions of Dye to Extract It at Room Temperature; Dye-Loaded Magnetic NPs are Collected with an External magnet and Used in Part (b); The Concentration of the Dye Left in the Solution is Measured with UV-Visible to Determine the Extracted Amount of Dye; (b) The Purified Dye-Loaded Magnetic NPs Are Analyzed with FTIR, XPS, TEM, FESEM, and EDS to Determine the Functionalities As Well As Elemental Composition of Both Dye and Surfactant/CD Involved in the Intermolecular Interactions Responsible for Extraction

In view of the depletion of freshwater resources, the choice of new sustainable technologies for cleaning contaminated water is the necessity of time. A large body of polluted untreated water around the world mainly originating from the textile industry^1,2^ flows into rivers and streams every year. It is possible to reuse this large body of contaminated water if treated appropriately with technologies based on functionalized nanomaterials.^3−5^ Recently, functionalized magnetic NPs have fetched enormous importance in environmental remediation.^6,7^ A variety of magnetic nanomaterials are used as dye absorbents and for wastewater treatment. Among them, biodegradation of wastewater dye,^8^ ion exchange, adsorption,^9^ and photocatalysis^10^ are the most common methodologies. The methodology based on magnetic nanoparticles (NPs) functionalized with surface active molecules, as well as macrocyclic compounds such as cyclodextrins (CDs) that can interact with water-soluble dyes through specific interactions is considered to be more precise and efficient for quantitative extraction of dye molecules. Surface active molecules like surfactants immobilized on the surface of magnetic NPs possess dual ability to interact with polar dyes through both hydrophilic as well as hydrophobic interactions,^11^ whereas surface-immobilized CD molecules act as an ideal harbor for accommodating the aromatic moieties of dye molecules. Thus, in this study, we choose to exploit the surfactant–dye^12,13^ and CD–dye^14,15^ interactions for the extraction of dye from contaminated water by using surfactant- and CD-functionalized magnetic NPs (Scheme 1a). In aqueous bulk, surfactant/CD molecules prefer to complex with the dye through specific interactions, which could be predominantly electrostatic or nonelectrostatic. If such interactions take place at the solid–liquid interface between the surfactant or CD molecules immobilized on the surface of magnetic NPs and aqueous bulk-solubilized dye, it is possible to extract dye from aqueous bulk simply by applying the external magnetic field. Thus, surfactant/CD functionalized magnetic NPs are highly promising vehicles for the extraction of dyes from contaminated water.

Both types of dye-responsive magnetic NPs can be easily synthesized following the hydrothermal synthesis which produces tiny magnetic NPs with a large surface area.^16^ The magnetic NPs thus produced have a high affinity to interact and complex with the aqueous bulk-solubilized dye molecules. The extraction ability of functionalized magnetic NPs depends on the strength of interactions operating between the surface-immobilized surfactant or CD molecules and the aqueous-solubilized dye (Scheme 1b). Stronger interactions allow rapid deposition of the dye on magnetic NPs that in turn facilitates extraction.^5,17,18^ Since the interactions between the dye and surfactant/CD molecules take place at the solid–liquid interface, molecular functionalities of surface-adsorbed surfactant/CD molecules play an important role in the extraction process.^12^ It can be optimized with the headgroup and/or hydrocarbon chain modifications of polar surfactant molecules. Introducing stronger polarity in the headgroup region of the polar surfactant is expected to have stronger interactions with polar dyes. Likewise, nonpolar CD cavity plays an important role for successful encapsulation and extraction of dyes by complexing with the nonpolar functional groups such as aromatic moieties.

The methodology presents a comprehensive and quantitative view of molecular functionalities for a successful solid–liquid interfacial extraction of a wide variety of dyes from aqueous bulk by using functionalized iron-oxide magnetic NPs. We expect that the findings drawn from this study will help in addressing the dye contamination issues arising from the large body of food and textile industries simply by using environmentally friendly functionalized magnetic NPs.

## Experimental Section

### Materials

Cetyltrimethylammonium bromide, (CTAB), hexadecyldimethyl-3-ammonio-1-propanesulfonate (HPS), hexamethylene bis(hexadecyl dimethylammonium bromide) (16–6–16),^16^ tris(2-(N-dodecyl *N*,*N*-dimethylammonio)ethylamine) tribromide (TriCAT),^16^ α, β, γ-CD, ferric chloride (FeCl_3_), ferrous sulfate (FeSO_4_), Allura Red AC, C_18_H_14_N_2_Na_2_O_8_S_2_ (Red 40), Tartrazine, C_16_H_9_N_4_Na_3_O_9_S_2_ (Yellow 5), Sunset yellow, C_16_H_10_N_2_Na_2_O_7_S_2_ (Yellow 6), Brilliant blue, C_37_H_34_N_2_Na_2_O_9_S_3_ (Blue 1), Coomassie Brilliant Blue G-250, C_47_H_48_N_3_NaO_7_S_2_ (CBB), Pyrogallol Red, C_19_H_12_O_8_S (P Red), Eriochrome black T, C_20_H_12_N_3_O_7_SNa (Black T), and Congo red, C_32_H_22_N_6_Na_2_O_6_S_2_ (C Red) were obtained from Aldrich. The molecular structure of all surfactants, dyes, and CDs used is shown in the Supporting Information, Figure S1a–d. Double distilled water was used for all of the preparations.

### Synthesis of Functionalized Iron-Oxide NPs

Surfactant/CD-functionalized iron-oxide NPs were synthesized by following the hydrothermal synthesis reported earlier.^16^ Typically, 20 mL of the reaction mixture containing 4 mM FeCl_3_ and 4 mM FeSO_4_ in a 1:1 mol ratio along with 100 mg of surfactant or 5 mM CD were taken in a Teflon container. CD was readily soluble in the aqueous salt solution, whereas dissolution of surfactant usually required little heat to dissolve. Addition of 50% ammonium hydroxide initiated the base-catalyzed reaction. The reaction mixture was sealed in a steel jacket and placed at a constant temperature of 150 °C for 24 h. After the completion of the reaction, surfactant- or CD-functionalized magnetic NPs were collected by using an external magnet and purified repeatedly from pure water to remove excess of surfactant/CD.

### Extraction

Extraction of dye molecules from aqueous dye solution was carried out by taking a constant amount of 40 mM purified magnetic NPs in a series of 3 mL of aqueous dye solutions of 0.05–8.0 mM as model systems (Scheme 1a). These solutions were placed at room temperature undisturbed with occasional shaking for at least 1 week to provide maximum time for extraction. Depending on the surface functionalities of magnetic NPs, extraction took from a few minutes to several hours to completely change the colored dye to colorless solution. After 1 week, dye-loaded magnetic NPs were removed by using an external magnet, and the absorbance of the remaining dye solution was measured to determine the extracted amount of dye. The dye-loaded magnetic NPs purified from pure water to remove excess amount of dye were analyzed for surface-adsorbed dye molecules (Scheme 1b). It is to be mentioned that unlike conventional NPs, magnetic NPs possess inherent ability to respond to an external magnetic field. Application of the external magnetic field ensures the removal of the maximum number of NPs without introducing any toxicity due to their leaching in the aqueous phase.

### Characterization

UV–visible measurements of dye solutions before and after the extraction were carried out by using a UV–visible instrument (Shimadzu-Model No. 2450, double beam) equipped with a TCC 240A thermoelectrically temperature-controlled cell holder. The shape, size, and elemental composition of dye-loaded magnetic NPs were characterized by using high-resolution transmission electron microscopy (TEM) with a Jeol 2010F field emission gun (FEG) operated at 200 kV. Field emission scanning electron microscopy (FESEM) analysis was performed by dispersing a drop of sample on a carbon tapped stub, coated with platinum, and then analyzed with a Hitachi SU8010-SE instrument of resolution 1.3 nm at a landing voltage of 1.0 kV. EDS analyses were done simultaneously with a Bruker eds model XFlash 6130 with a peak resolution of 121 eV. Infrared absorption measurements were recorded with a FTIR spectrometer (Shimadzu) in the range of 4000–400 cm^–1^. Each spectrum was measured in the transmission mode with 256 scans and 4 cm^–1^ resolution. The surface analysis of each sample was carried out using a Kratos Axis Ultra X-ray photoelectron spectrometer (XPS). The samples were analyzed at 5% power with an exposure time of 30 s, accumulated four times. A 50× objective was used to focus the laser onto the surface, and the spot size analyzed was ∼1 μm in diameter.

## Results and Discussion

The work is hypothesized on the basis of intermolecular interactions operating between different functionalities immobilized at the solid–liquid interface of magnetic NPs and aqueous bulk-solubilized dye molecules. That drives the complexation and deposition of the dye on the magnetic NPs, thereby removing them from the contaminated water under the effect of an external magnetic field. In order to accomplish this, a simple and efficient protocol is adopted where extraction is first observed with the naked eye at room temperature followed by quantitative measurements with UV–visible spectroscopy. From UV–visible studies, the amount of dye extracted was quantitatively evaluated and compared for different functionalized magnetic NPs to determine the functionalities responsible for the efficient removal of the dye.

### Surfactant-Functionalized Magnetic NPs

**Figure 1:**
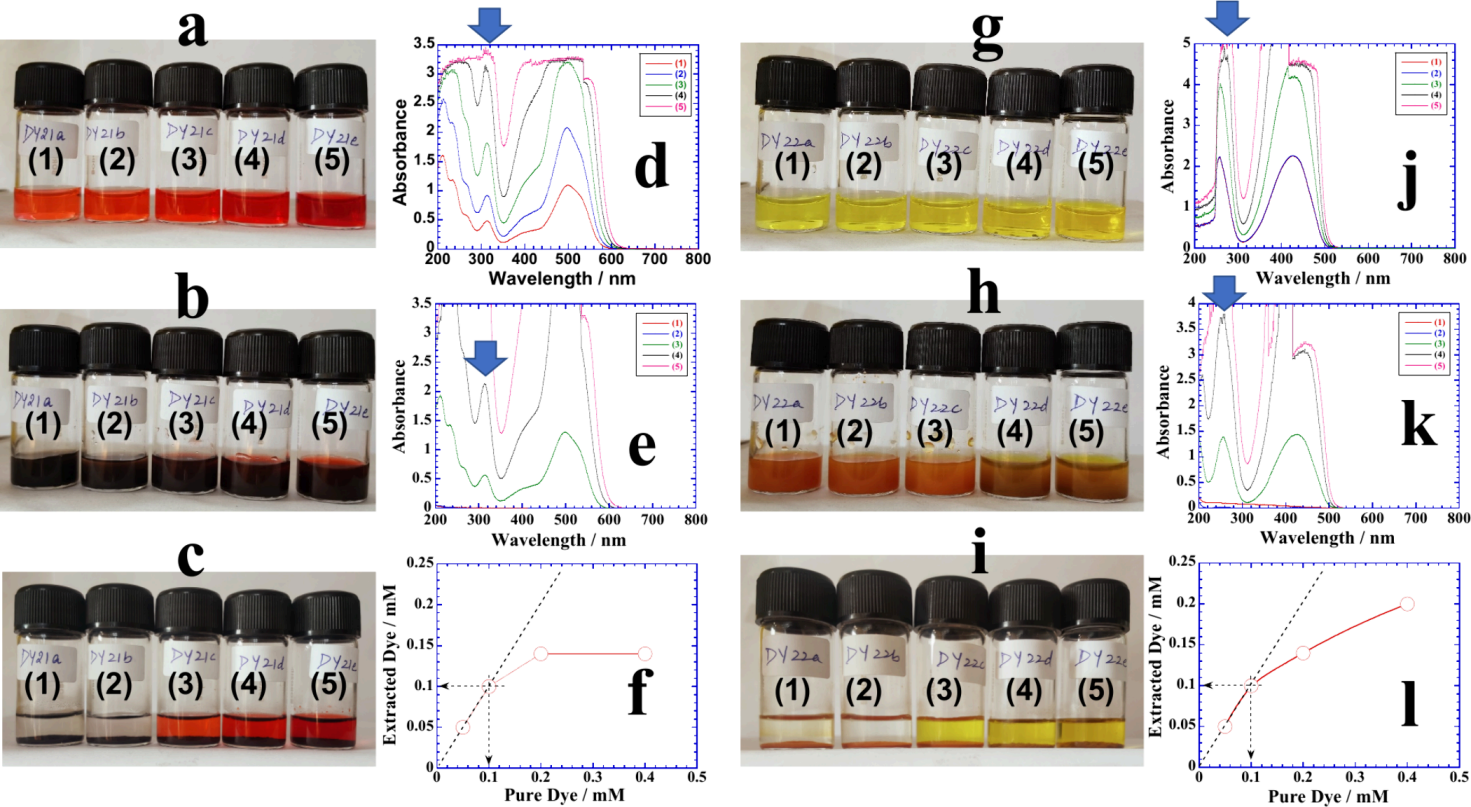
(a) Photos of aqueous Red 40 solutions of (1) 0.05, (2) 0.1, (3) 0.2, (4) 0.4, and (5) 0.8 mM. (b) Photos after the addition of 40 mM 16–6–16-functionalized magnetic NPs in each bottle. (c) Photos after 1 week. (d, e) UV–visible spectra of the solution of each sample bottle of (a) and (c), respectively. (f) Plot of extracted Red 40 versus the amount before the addition of magnetic NPs. (g) Photos of aqueous Yellow 5 solutions of (1) 0.05, (2) 0.1, (3) 0.2, (4) 0.4, and (5) 0.8 mM. (h) Photos after the addition of 40 mM 16–6–16-functionalized magnetic NPs in each bottle. (i) Photos after 1 week. (j, k) UV–visible spectra of the solution of each sample bottle of (g) and (i), respectively. (l) Plot of extracted Yellow 5 versus the amount before the addition of magnetic NPs.

**Figure 2:**
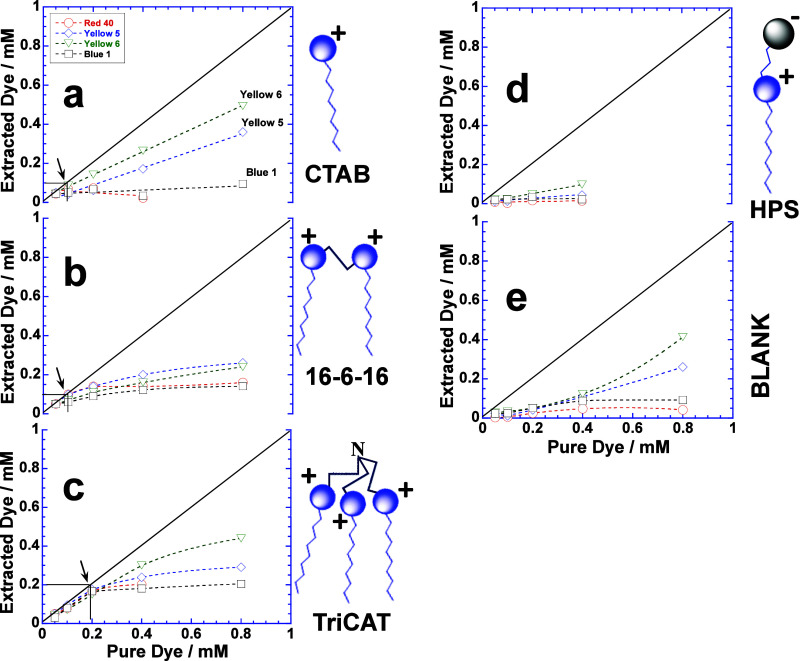
Plots of extracted dyes (Red 40, Yellow 5, Yellow 6, and Blue 1) versus the amount of pure dye in aqueous solution. Extraction performed with (a) CTAB, (b) 16–6–16, (c) TriCAT, (d) HPS, and (e) blank magnetic NPs. Black arrows indicate the “maximum concentration” of each dye extracted leaving behind a colorless solution.

**Figure 3:**
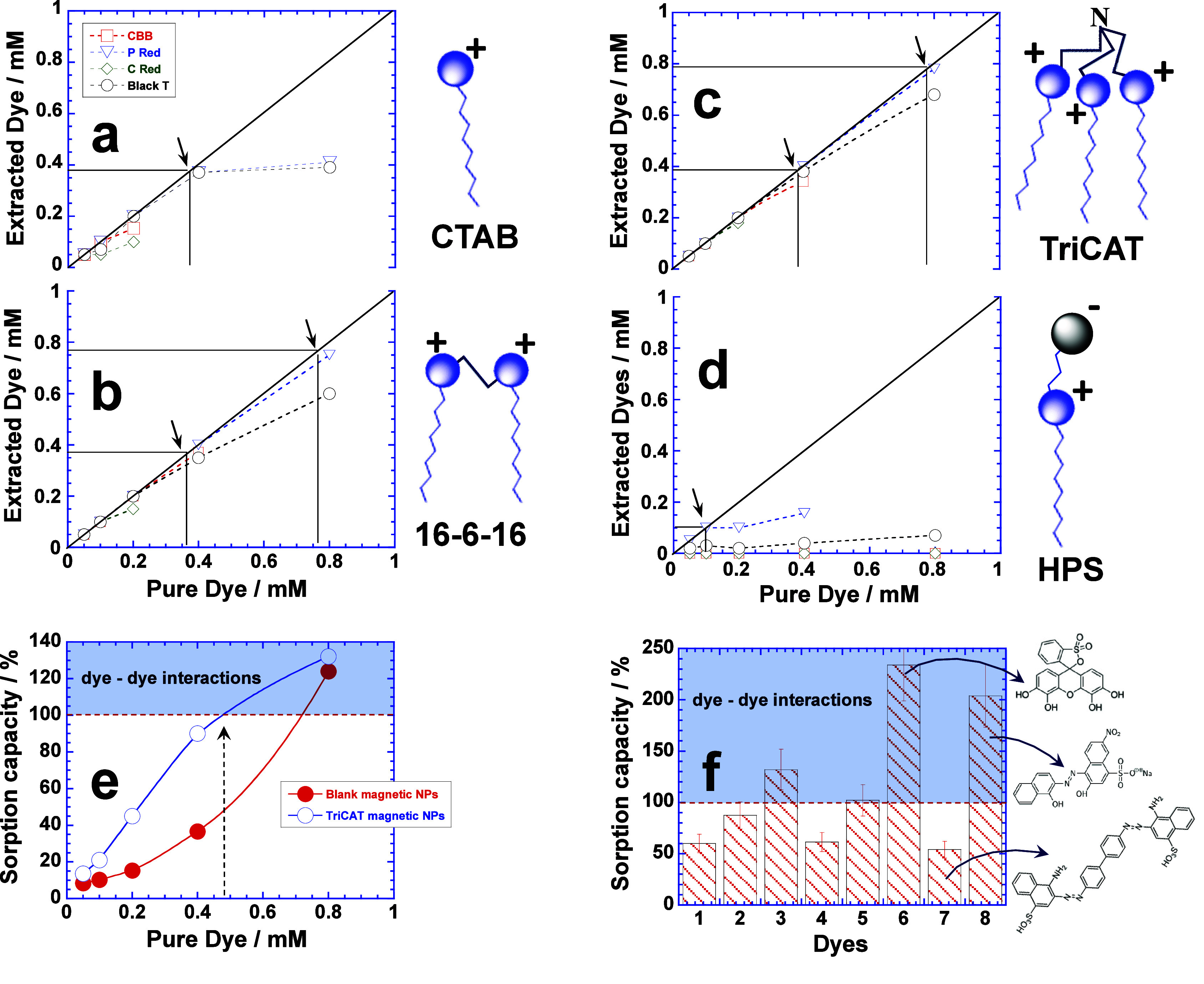
Plots of extracted dyes (CBB, P Red, C Red, and Black T) versus the amount of pure dye in aqueous solution. Extraction performed with (a) CTAB, (b) 16–6–16, (c) TriCAT, and (d) HPS functionalized magnetic NPs. Black arrows indicate the “maximum concentration” of each dye extracted leaving behind a colorless solution. Comparison between the sorption capacities of blank and TriCAT-functionalized magnetic NPs for Yellow 6 (e). Bar plots (f) of sorption capacity of TriCAT-functionalized magnetic NPs for various dyes (1, Red 40; 2, Yellow 5:3, Yellow 6; 4, Blue 1; 5, CBB; 6, P Red; 7, C Red; 8, Black T). Shaded areas in (e) and (f) represent the sorption capacity exceeding 100% due to dye–dye interactions.

Figure 1a–f,g–l shows typical extractions of Red 40 and Yellow 5, respectively, from a series of aqueous solutions by treating them with 40 mM 16–6–16 functionalized magnetic NPs. The first two concentrations of the dye in both cases are completely extracted leaving behind colorless solutions. The amount of dye extracted is determined from UV–visible measurements (Figure 1d–f,j–l, respectively) of dye solutions without and with magnetic NPs, and it is plotted against the initial concentration of Red 40 (Figure 1f) and Yellow 5 (Figure 1l). Similar extraction experiments are performed with CTAB, TriCAT, and HPS functionalized magnetic NPs (Figures S2–S5), and the amount of dye extracted is shown in Figure 2. Since all dyes are charged molecules, their extraction is tested with cationic (Figure 2a–c) and zwitterionic (Figure 2d, results are not clear beyond 0.4 mM dye due to turbidity) functionalized magnetic NPs, whereas extraction with anionic sodium dodecyl sulfate-functionalized magnetic NPs is not performed because they are water-insoluble.^16^ Increase in the positive charge of cationic surfactants (i.e., CTAB < 16–6–16 < TriCAT) due to the number of cationic head groups facilitates the extraction (Figure 2a–c). The “maximum extraction” (that makes the solution colorless) is indicated by arrows which represent the extraction of the entire dye, i.e., 0.2 mM (Figure 2c) and 0.1 mM (Figure 2a,b) by TriCAT and CTAB/16–6–16 functionalized magnetic NPs, respectively, whereas it is least with HPS-functionalized magnetic NPs (Figure 2d). Among all dyes, Blue 1 is the least extracted, whereas Yellow 5 and 6 show maximum extraction. Another interesting aspect of Figure 2 is that the extraction continues to rise in each case before tending to be constant. This is not expected if magnetic NPs get saturated with the dye at “maximum extraction,” but could happen only if dye molecules adsorbed on the surface of magnetic NPs are further involved in the dye–dye interactions.^19,20^ Both Yellow 5 and 6 depict maximum dye–dye interactions where extraction keeps on increasing with the concentration of the dye and is more prominent with CTAB and TriCAT functionalized magnetic NPs. In contrast, when extraction is performed with bare magnetic NPs, no extraction takes place within the 0.2 mM concentration range of the dye, but it starts thereafter (Figure 2e). It further supports our earlier inference that the dye adsorbed at the solid–liquid interface of magnetic NPs facilitates the dye–dye interactions. Here too, Yellow 5 and 6 show maximum extraction due to dye–dye interactions. Similar extractions are performed with another set of dyes, i.e., CBB, P Red, C Red, and Black T, and the results are presented in Figure 3 (to avoid overcrowding of Figure 2). These dyes show better extraction in comparison to that shown in Figure 2. CTAB-stabilized magnetic NPs (Figure 3a) extract almost a 4 times greater amount of these dyes in comparison to that of Figure 2. Likewise, extraction is much more rapid and facilitated by 16–6–16 and TriCAT functionalized magnetic NPs (Figure 3b,c, respectively), whereas it is minimum for HPS-functionalized magnetic NPs (Figure 3d). Here, P Red and C Red depict much facilitated extraction in comparison to CBB and Black T.

The “maximum extraction” values for all dyes with different surfactant-functionalized magnetic NPs are compared in Table S1. Both 16–6–16 and TriCAT functionalized magnetic NPs are promising functionalized magnetic NPs with much greater extraction efficiency in comparison to those of CTAB and HPS functionalized magnetic NPs. Among the dyes, the extraction of smaller molecular mass dyes seems to be facilitated in comparison to the dyes with a larger molecular mass.^21,22^ Thus, stronger polarity of the surfactant (16–6–16 or TriCAT) adsorbed on the surface of magnetic NPs drives favorable extraction of the dye with a smaller molecular size (yellow 6 or P Red) because the solid–liquid interfacial adsorption of smaller dye molecules is less prone to steric constraints.^16^ The extraction of dyes are further quantified on the basis of sorption capacity which is the amount of dye taken up by the functionalized magnetic NPs per unit mass.^23,24^Figure 3e compares the sorption capacities of TriCAT-functionalized and blank magnetic NPs of Yellow 6 over a concentration range of 0–1 mM. The sorption capacity is obviously much greater for TriCAT than for blank NPs, but the dye–dye interactions predominate beyond 0.4 mM of Yellow 6 (see dotted arrow, Figure 3e) for the TriCAT-functionalized magnetic NPs that exceed the sorption capacity by more than 100%. However, higher concentration of Yellow 6 (i.e., 8 mM) also induces the dye–dye interactions for blank magnetic NPs, and as a result, their sorption capacity approaches that of TriCAT-functionalized magnetic NPs. Thus, the predominance of the dye–dye interactions in fact overshadows the extraction ability of functionalized magnetic NPs, producing little difference between functionalized and blank magnetic NPs. Figure 3f compares the sorption capacity of TriCAT-functionalized magnetic NPs for all dyes. Interestingly, it exceeds 200% for P Red and Black T, whereas it is close to just 50% for C Red, further authenticating the facilitated extraction by dye–dye interactions. The meaning of 200% sorption capacity is best explained in terms of strong dye–dye interactions, which allows the adsorption of a large amount of dye on the surface of magnetic NPs.

### CD-Functionalized Magnetic NPs

**Figure 4:**
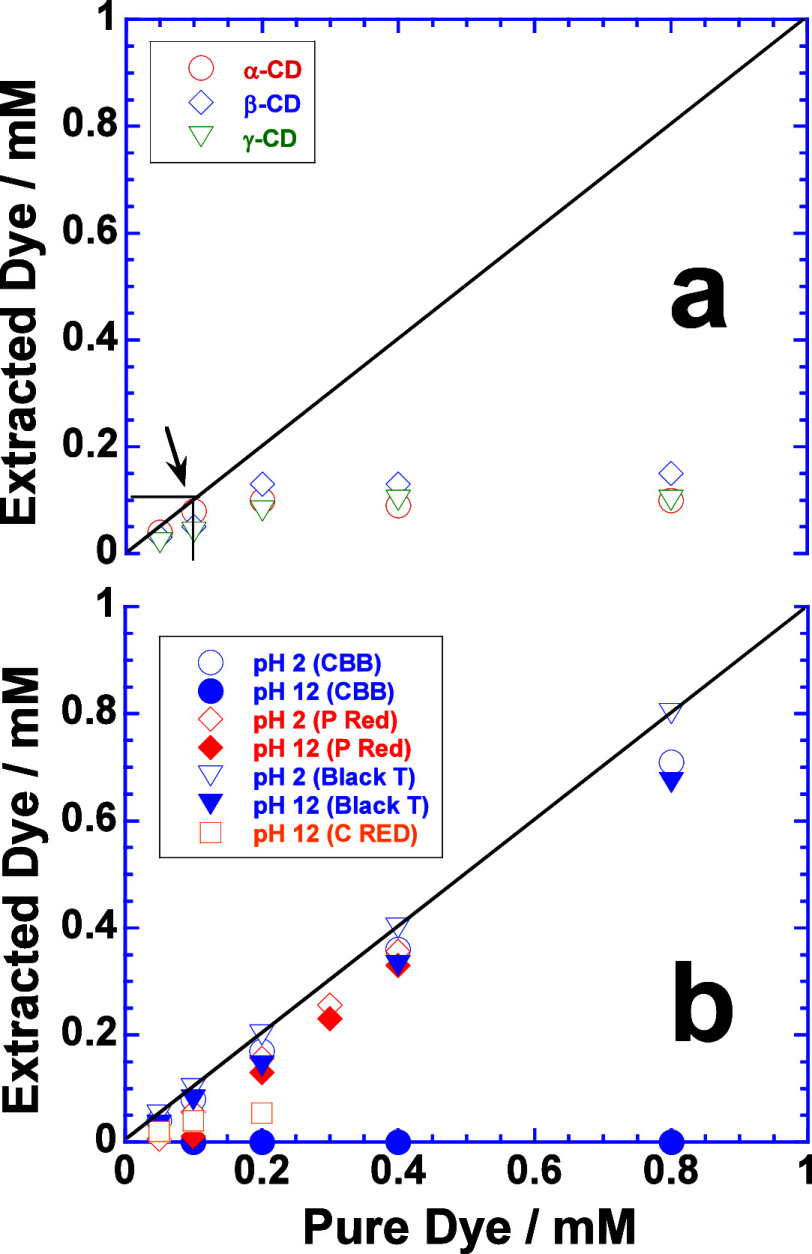
Plots of extracted dyes CBB versus the amount of pure CBB in aqueous solution. Extraction performed with (a) α-CD, (b) β-CD, and (c) γ-CD functionalized magnetic NPs. Black arrow indicates the “maximum concentration” of CBB extracted leaving behind a colorless solution.

Dyes are also known to interact and encapsulate in the nonpolar cavity of CD macrocyclic compounds.^14,15^ The colored dyes usually consist of aromatic functionalities which act as optimal guests for the nonpolar cavity of CD.^14^ Such encapsulation is also expected to occur at the solid–liquid interface of magnetic NPs that are functionalized with CD molecules.^25,26^ Thus, CD molecules immobilized on the surface of magnetic NPs encapsulate and entrap bulk-solubilized dye molecules and hence act as ideal vehicles for their extraction. The extraction of CBB with α-, β, and γ-CD stabilized magnetic NPs is shown in Figure 4, whereas similar extraction is observed of P Red, C Red, and Black T (Figures S6 and S7). At neutral pH, CD-stabilized magnetic NPs extract only 0.1 mM dye, and that too does not depend on the cavity size of α-, β-, and γ-CD (Figure 4a). It shows that the host–guest interactions between the aqueous solubilized dye (guest) and CD cavity (host) are not significant enough to promote extraction. Immobilization of CD on the surface of magnetic NPs impedes the degree of freedom of CD cavity and induces steric constraints for appropriate host–guest interactions.^26^ The latter complexation requires a proper orientation of the CD cavity to incorporate the dye molecule which is not achieved in the neutral medium. However, the extraction of CBB increases significantly at pH 12, whereas no extraction occurs at pH 2. Both P Red and Black T are extracted at low and high pH, but C Red does not show any marked extraction. Such a facilitated extraction under the effect of pH indicates the participation of host–guest interactions where the CD cavity prefers to entrap predominantly nonpolar functional groups of dye molecules.^27−29^ It happens when polarity is induced in organic dye molecules by either high or low pH that in turn promotes their solid–liquid interfacial adsorption. The latter effect facilitates the encapsulation of aromatic moiety of dye in the CD cavity immobilized on the solid–liquid interface of magnetic NPs.^28^ For instance, both P Red and Black T can be protonated at low pH and deprotonated at high pH due to the presence of −N=N– and/or −OH functional groups. This facilitates their interfacial adsorption, thus allowing aromatic moieties to be involved in host–guest interactions with the CD cavity and hence promoting the extraction.^29^

### Relative Comparison between Surfactant and CD Functionalized Magnetic NPs

In neutral medium, surfactant-functionalized magnetic NPs perform much better extraction (Figures 2 and 3) in comparison to CD-functionalized magnetic NPs (Figure 4a). However, at low or/and high pH, CD-functionalized magnetic NPs are excellent extractors (Figure 4b). The pH effect is insignificant for surfactant-functionalized magnetic NPs due to their ionic character. All surfactant-functionalized magnetic NPs show a greater extraction of Yellow 5 and 6. The facilitated extraction of most of the dyes under the effect of pH by CD-functionalized magnetic NPs is further related to the self-association of dye molecules. The self-association is expected to be more pronounced close to neutral pH where hydrophobic interactions among the dye molecules are predominantly higher in comparison to the hydrophilic interactions.^29^ However, a low or high pH reduces the self-association by enhancing the ionic character of the dye molecule, thereby generating monomeric species with favorable host–guest interactions to form inclusion complexes with CD-stabilized magnetic NPs and hence facilitating the extraction.

### IR Analysis

**Figure 5:**
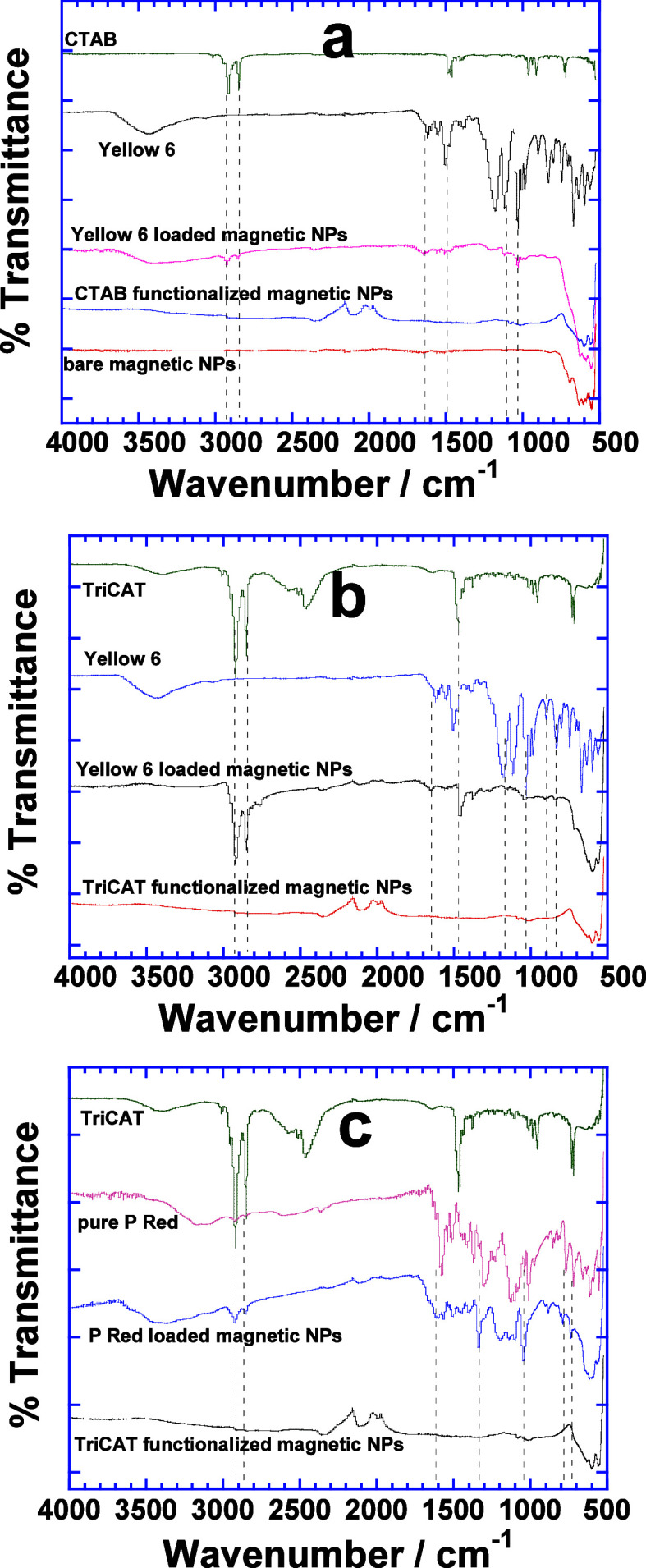
FTIR spectra of Yellow 6-loaded CTAB (a) and TriCAT (b) functionalized magnetic NPs. (c) IR spectra of P Red-loaded TriCAT-functionalized magnetic NPs.

The extraction of the dye is further analyzed by performing the IR analysis of dye-loaded functionalized magnetic NPs in the dried state (Figure 5). Bare magnetic NPs show two characteristic peaks between 580 and 630 cm^–1^ due to the stretching vibrational modes of Fe–O bonds in crystalline Fe_3_O_4_ NPs.^30^ CTAB-functionalized magnetic NPs (Figure 5a) provide a relatively broad IR band at 2115 cm^–1^ and a weak band at 1994 cm^–1^ due to Fe–C and Fe–CO stretching, respectively,^31^ whereas other characteristic bands of C–H, C–C, and C–N functional groups are not observed due to the hydrothermal treatment at an elevated temperature and pressure. However, the IR spectrum of Yellow 6-loaded magnetic NPs clearly depicts the vibrational bands due to (−C–N−)_str_, (−SO_3_-)_str_, (−N=N−)_str_, (−N–H−)_bend,_ and (C–C ring)_str_ at 1030, 1117, 1652, 1558, and 1503 cm^–1^, respectively, of Yellow 6.^32^ Apart from this, vibrational modes of the hydrocarbon chain of CTAB due to (−CH_3_)_asym_ and (−CH_2_)_sym_ at 2924 and 2829 cm^–1^, respectively, are also evident.^33^ Interestingly, these modes remain hidden in CTAB-functionalized magnetic NPs probably due to hydrothermal treatment. However, they become prominent when treated with aqueous dye because the surface-adsorbed surfactant interacts with dye molecules in the aqueous bulk.

Similarly, the IR spectra of Yellow 6-loaded TriCAT-functionalized magnetic NPs (Figure 5b) also show the vibrational bands of Yellow 6 due to (−N–H)_wag_ at 898 and 831 cm^–1^, (−C–N−)_str_ at 1041 cm^–1^, (−SO_3_−)_str_ at 1176 cm^–1^, and (−N–H−)_bend_ at 1653 cm^–1^. This spectrum also shows the vibrational bands of TriCAT^33^ due to (CH_2_)_sci_ at 1457 cm^–1^ as well as (CH_2_)_asym_ at 2919 cm^–1^ and (CH_2_)_sym_ at 2850 cm^–1^ which represent the surface-adsorbed hydrocarbon chains of TriCAT. On the other hand, the extraction of P Red with TriCAT-functionalized magnetic NPs demonstrates much stronger adsorption (Figure 5c) and is evident from the vibrational bands of (C–H)_bend_ at 789 and 736 cm^–1^, whereas (ring deformation)_in-plane bend_ at 1045 and (C–O)_str_ at 1334 cm^-1^are blue-shifted in comparison to their vibrational modes in pure P Red.^34^ This unique behavior of adsorption of P Red at the solid–liquid interface is quite different from that of Yellow 6 where no blueshift in the vibrational modes of Yellow 6 is observed. Table S2 lists the vibrational modes of these samples.

The blueshift in the vibrational modes of ring deformation of P Red highlights the facilitated adsorption at the solid–liquid interface of TriCAT-functionalized magnetic NPs (Figure 5c). That originates from the interactions of tricationic head groups of TriCAT with −OH functional groups of P Red^35^ and is the consequence of maximum extraction among all dyes (Table S1). In addition, P Red also shows little dye–dye interactions even at higher concentrations which is contrastingly different from Yellow 6 where dye–dye interactions are quite prominent (compare Figures 2 and 3). Thus, ring deformation depicts the adsorption of P Red on the surface of magnetic NPs without undergoing dye–dye interactions. In contrast, IR analysis is unable to detect the ring deformation of Yellow 6 upon its interactions with the tricationic headgroup of TriCAT because they are overshadowed by the dye–dye interactions (Figure 2).

### XPS Analysis

**Figure 6:**
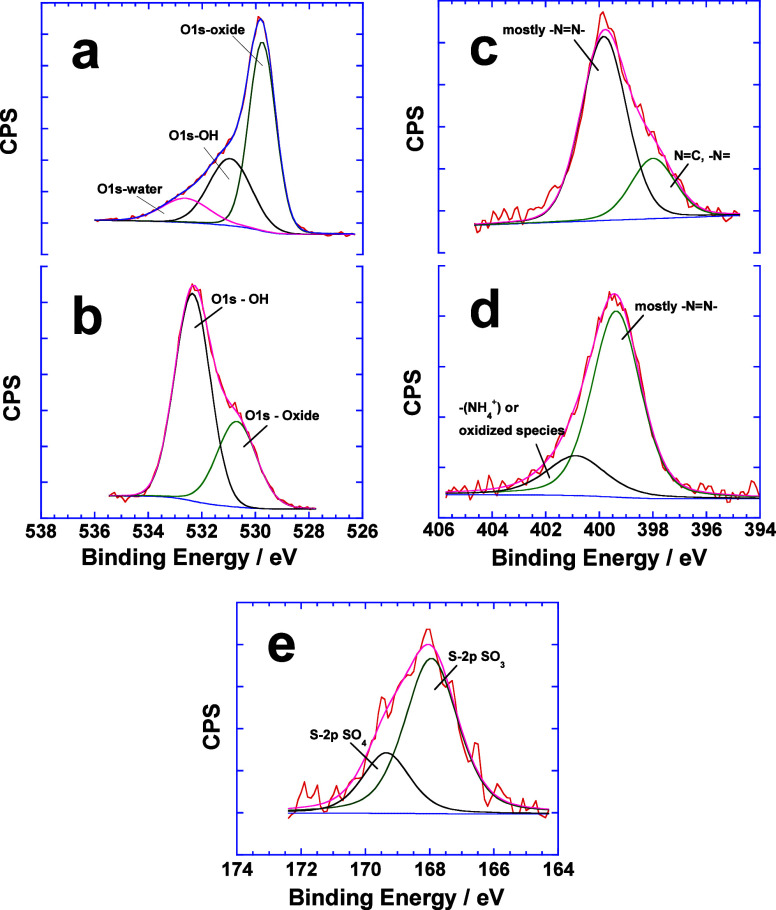
High-resolution XPS spectra for O 1s (a, b), N 1s (c, d), and S 2p (e). See details in the text.

XPS analysis is the most appropriate semiquantitative technique to determine the different functional groups of the extracted dye adsorbed on the surface of magnetic NPs. Two oxidation states of Fe 2p (Figure S8) in FeO and Fe_2_O_3_ with greater relative abundance of Fe^3+^ in comparison to Fe^2+^ constitute iron-oxide magnetic NPs and hence confirm the Fe_3_O_4_ composition.^36^ Since iron-oxide NPs are crystalline and monodispersed particles (TEM analysis in the next section), extraction and adsorption of the dye do not alter this composition, and the binding energies of both oxidation states (Fe^3+^ as well as Fe^2+^) remain the same in the absence and presence of the dye. The binding energies of O 1s provide important information about the adsorption of the dye. The binding energy of 529.36 eV^37^ refers to the presence of metal oxide due to Fe_3_O_4_ magnetic NPs whose surface area depends on the amount of extracted dye. For both Red 40 and Blue 1 loaded magnetic NP samples, the amount of metallic oxide is maximum (Figure 6a), whereas it is much smaller than that of the O 1s hydroxide for Yellow 6 and 5 (Figure 6b). Thus, greater deposition of the dye on the surface of magnetic NPs increases the concentration of O 1s hydroxide in the sample.

Similarly, the binding energy of N 1s provides some interesting information. Since most of the dyes studied in this work are azo dyes, a binding energy around 399.5 eV^38,39^ with maximum surface area due to −N=N- is prominently observed in these cases (Figure 6c). A much smaller peak with binding energy around 398 eV^40,41^ is attributed to the nitrogen species in the chemical environment where they are primarily bonded to carbon atoms as in the case of Blue 1. Apart from this, most of the samples also show another N 1s peak between 401 and 402 eV (Figure 6d) due to ammonium^42,43^ functional groups of 16–6–16 or due to the presence of oxidized nitrogen species where instead of 16–6–16, CD-functionalized magnetic NPs are used. The XPS spectrum of S 2p (Figure 6e) provides independent evidence of the extraction and adsorption of various dyes on the surfaces of functionalized magnetic NPs. Binding energy of sulfonate functional groups around 167.9 eV^44^ is only due to the presence of dye molecules because this functional group is absent in all functionalized magnetic NPs used for extraction. Unfortunately, the low atom % of S 2p cannot be used for the qualitative extraction among various dyes because of the semiquantitative nature of XPS. Nevertheless, it clearly proves the extraction of all dyes by magnetic NPs.

The binding energies of various species and their atom % for different samples of extracted dyes are listed in Tables 1 and 2 for the surfactant and CD functionalized magnetic NPs, respectively. The amount of C 1s is maximum for all samples and is obviously expected from its presence in the molecular structures of all dyes as well as surfactant molecules (Table 1); but this is not true for the samples of CD-functionalized magnetic NPs (Table 2) because of the presence of comparable numbers of oxygen and carbon atoms in the molecular structure of CD. Here, the amount of O 1s is maximum followed by C 1s. In both tables, the amounts of C 1s and O 1s seem to be higher for P Red in comparison to those for other dyes, suggesting a facilitated extraction as observed from UV–visible studies.

**Table 1:** XPS Analysis of Dye-Loaded 16–6–16-Functionalized Magnetic NPs^a^

		dye-loaded functionalized magnetic NP									
		16–6–16–Red 40		16–6–16–Yellow 5		16–6–16–Yellow 6		16–6–16–Blue 1		16–6–16–P Red	
elements^b^		atom %	area %	atom %	area %	atom %	area %	atom %	area %	atom %	area %
O 1s	O 1s-oxide	34	59.2 (529.5)	35	8.9 (530.7)	38	35.1 (530.7)	36	53.1 (529.5)	38	42.6 (529.3)
	O 1s-water		8.3 (532.6)		67.1 (532.1)				24.6 (530.9)		20.1 (530.6)
	O 1s–OH		33.6 (530.7)				95.9 (532.4)		6.5 (532.3)		5.5 (532.3)
C 1s	C–C, C–H	45	44.5 (284.7)	47	65.5 (284.8)	48	42.3 (284.7)	47	49.2 (284.8)	49	59.9 (284.8)
	C–OH, C–O–C		7.3 (286.)		7.1 (286.0)		1.8 (286.1)		10.1 (286.1)		5.8 (285.8)
	C=O		1.1 (288.0)		2.9 (288.2)		0.8 (288.0)		2.2 (287.9)		0.9 (287.7)
Fe 2p	Fe 2p3/2 FeO	17	45.4 (709.9)	14	38.4 (709.2)	11	30.4 (709.3)	15	16.4 (708.9)	12	36.8 (709.8)
	Fe 2p1/2 FeO		22.7 (722.9)		11.9 (723.1)		12.2 (722.9)		8.2 (723.2)		18.4 (723.4)
	Fe 2p3/2 Fe_2_O_3_		55.1 (712.0)		45.1 (712.1)		34.1 (712.3)		45.6 (711.1)		14.6 (711.9)
	Fe 2p1/2 Fe_2_O_3_		27.5 (724.7)		17.5 (724.1)		21.5 (724.4)		22.8 (724.2)		7.3 (725.9)
	Fe 2p3/2 satellite		15.6 (718.5)		25.6 (718.8)		11.6 (718.1)		4.4 (718.8)		3.7 (718.4)
	Fe 2p1/2 satellite		6.9 (732.1)		8.9 (732.0)		11.9 (732.3)		2.4 (732.2)		6.2 (732.2)
N 1s	N=C, −N=	3	17.4 (399.5)	2	6.5 (397.9)	1	6.3 (398.3)	1	10.9 (399.2)	2	11.24 (398.6)
	NC_3_, −NH_2_,		11.5 (402.1)		19.9 (399.8)		9.7 (399.8)		7.1 (400.1)		
S 2p	S 2p-SO_3_	1	14.1 (167.5)	1	7.1 (167.1)	1	8.2 (167.3)	1	10.4 (167.5)		
	S 2p-SO_4_		3.6 (169.0)		4.3 (168.9)		4.1 (169.0)		5.6 (168.7)		

a Atom % and binding energies/eV are listed in parentheses in the same order in a column below the area %.

b Na-1s data are omitted due to its very low amount in the sample.

**Table 2:** XPS Analysis of Dye-Loaded CD-Functionalized Magnetic NPs^a^

		dye loaded functionalized magnetic NP											
		α-CD–CBB		β-CD–CBB		γ-CD–CBB		β-CD–Black T		β-CD–C Red		β-CD–P Red	
elements^b^		atom %	area %	atom %	area %	atom %	area %	atom %	area %	atom %	area %	atom %	area %
O-1s	O 1s-oxide	44	89.2 (529.7)	43	58.8 (529.8)	43	61.7 (529.8)	48	25.5 (531.3)	46	74.5 (529.9)	48	77.9 (529.8)
	O 1s-water		31.5 (532.2)		20.9 (532.5)		24.3 (532.7)		24.5 (532.7)		21.9 (532.6)		33.8 (531.8)
	O 1s–OH		28.8 (530.9)		48.1 (530.7)		44.6 (530.9)		67.4 (530.0)		34.4 (530.8)		18.5 (530.9)
	O 1s–C-O												16.1 (533.0)
C-1s	C–C, C–H	28	30.3 (284.6)	28	39.1 (284.6)	29	40.8 (284.6)	24	53.3 (284.6)	25	30.2 (284.6)	26	42.6 (284.7)
	C–OH, C–O–C		27.1 (285.9)		31.9 (285.9)		31.2 (285.8)		28.8 (285.9)		17.0 (286.1)		21.6 (286.0)
	C=O		10.4 (288.0)		9.7 (288.1)		13.4 (287.9)		14.7 (288.0)		10.5 (287.9)		12.1 (288.1)
Fe-2p	Fe 2p3/2 (metal)	25	4.9 (707.9)	26	3.2 (708.0)	25	3.8 (708.0)	23	2.7 (708.3)	26	3.3 (707.9)	23	4.6 (707.9)
	Fe 2p3/2 (FeO)		67.3 (710.1)		64.5 (710.2)		58.6 (710.3)		49.3 (710.4)		62.1 (710.3)		60.0 (710.2)
	Fe 2p3/2 Fe_2_O_3_		82.1 (711.9)		60.4 (712.4)		59.5 (712.4)		57.3 (712.3)		83.7 (712.3)		63.4 (712.2)
	Fe 2p1/2 Fe_2_O_3_		35.4 (723.6)		33.9 (723.6)		30.8 (723.7)		25.9 (723.6)		32.6 (723.5)		35.6 (723.5)
	Fe (II) 2p3/2 satellite		71.7 (718.5)		38.4 (718.7)		43.9 (718.5)		31.9 (718.7)		41.7 (718.7)		30.3 (718.6)
	Fe (III)2p1/2 satellite		23.9 (731.7)		14.5 (731.7)		19.5 (731.7)		15.9 (732.4)		21.2 (731.8)		14.9 (732.0)
	Fe 2p1/2 (FeO)		2.5 (722.5)		1.6 (721.6)		1.9 (721.8)		1.4 (721.2)		1.7 (721.2)		2.3 (721.8)
	Fe (II) 2p1/2 satellite		41.0 (725.9)		30.2 (725.9)		29.7 (725.9)		28.7 (725.9)		41.9 (725.8)		31.7 (725.9)
N-1s	N=C, −N=	2	17.3 (399.6)	2	19.2 (399.4)	2	19.9 (399.4)	4	21.7 (399.5)	2	16.9 (399.5)	3	25.8 (399.6)
	NC_3_, −NH_2_,		1.2 (402.4)		5.4 (400.9)		5.4 (400.6)		8.7 (400.9)		1.5 (402.5)		3.4 (402.5)
S-2p	S 2p-SO_3_	1		1	10.8 (168.0)	1	14.2 (167.9)	1	18.3 (167.9)				
	S 2p-SO_4_		5.2 (168.2)		3.8 (169.8)		5.9 (169.5)		6.4 (169.4)				

a Atom % and binding energies/eV are listed in parentheses in the same order in a column below the area %.

b Na-1s data are omitted due to its very low amount in the sample.

### Microscopy

**Figure 7:**
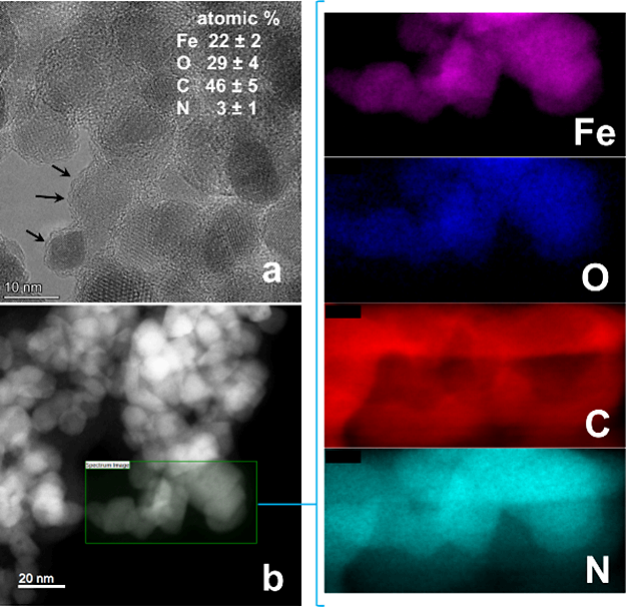
High-resolution bright-field (a) and low-resolution HAADF (b) TEM images of ∼10 nm size 16–6–16-functionalized iron-oxide NPs. Frames Fe, O, C, and N represent the elemental mapping. Black arrows in (a) show the thin film coating of 16–6–16 on the surface of magnetic NPs.

Before discussing the shape, structure, and elemental composition of dye-loaded magnetic NPs, it is important to understand the morphology of surfactant-functionalized magnetic NPs because they act as vehicles for dye extraction from the aqueous bulk. Figure 7 shows the electron energy loss spectroscopy (EELS) analysis of 16–6–16-functionalized magnetic NPs. EELS provides better insight into the surface analysis when NPs are coated with thin fluid film, and hence, it is best suited for determining the chemical composition of 16–6–16-functionalized magnetic NPs. Figure 7a,b shows the high-resolution bright-field and low-resolution high-angle annular dark-field (HAADF) TEM images of ∼10 nm size 16–6–16 functionalized iron-oxide NPs. They are clearly coated with a thin layer of about 2 nm in thickness (Figure 7a) which is in fact constituted by the bilayer of 16–6–16 (indicated by arrows).^16^ This surface-adsorbed bilayer is instrumental in initiating interactions with aqueous bulk-solubilized dye molecules. The chemical composition and elemental mapping are shown in the respective Fe, O, C, and N frames. Fe and O mapping is due to the iron-oxide NPs, whereas a thick layer of coating in the C frame is due to C which is mainly contributed by the hydrocarbon chains of 16–6–16 constituting the bilayer formation. It is important to note that the surface area constituted by N is almost the same as that of C because each hydrocarbon chain is further connected to a tetraalkylammonium headgroup of 16–6–16. The relative abundance in atom % for different elements is listed in Figure 7a.

**Figure 8:**
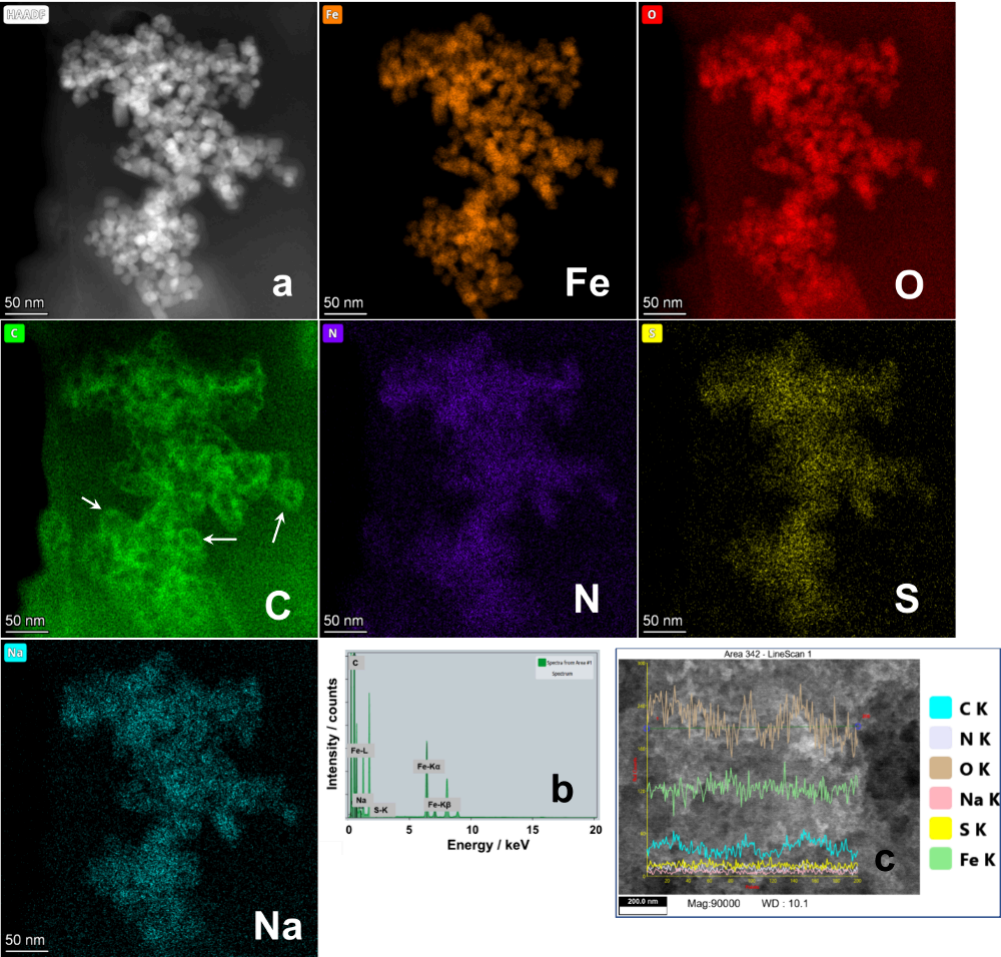
(a) HAADF TEM images of Yellow 6-loaded 16–6–16-functionalized iron-oxide NPs. Frames Fe, O, C, N, S, and Na represent the elemental mapping. (b) EDS spectrum of various elements. (c) Line analysis of the FESEM image of the same sample.

Figure 8 shows the microscopic analysis of Yellow 6-loaded 16–6–16-functionalized magnetic NPs of the sample (Figure S2b5). The HAADF image shows clear faceted magnetic NPs (Figure 8a) of ∼10 nm in size, whereas EDS mapping of Fe, O, C, N, S, and Na is shown in their respective frames. The faceted NPs are made up of Fe and O constituting the iron-oxide NPs. They are coated with C in the form of ring structures clearly visible in the C frame (indicated by arrows). The C mapping is collectively constituted by the methylene groups of 16–6–16 as well as aromatic moieties of Yellow 6. N mapping also originates from the surface area occupied by C due to ammonium and −N=N– functional groups of 16–6–16 and Yellow 6, respectively. The presence of S and Na mapping on the surface of magnetic NPs only originates due to sulfonate functional groups and their Na^+^ counterions of Yellow 6 authenticating the extraction of Yellow 6 by 16–6–16-functionalized magnetic NPs (EDS spectrum, Figure 8b). FESEM analysis (Figure S9) further supplements these results. EDS line analysis of the FESEM image covers a much larger surface area than TEM and clearly shows the presence of N, S, and Na in a much smaller amount in comparison to Fe, O, and C (Figure 8c). Similar analysis is performed for the Red 40-loaded 16–6–16-functionalized magnetic NPs of sample (Figure 1c5), and the detailed TEM analysis is shown in Figure S10.

**Figure 9:**
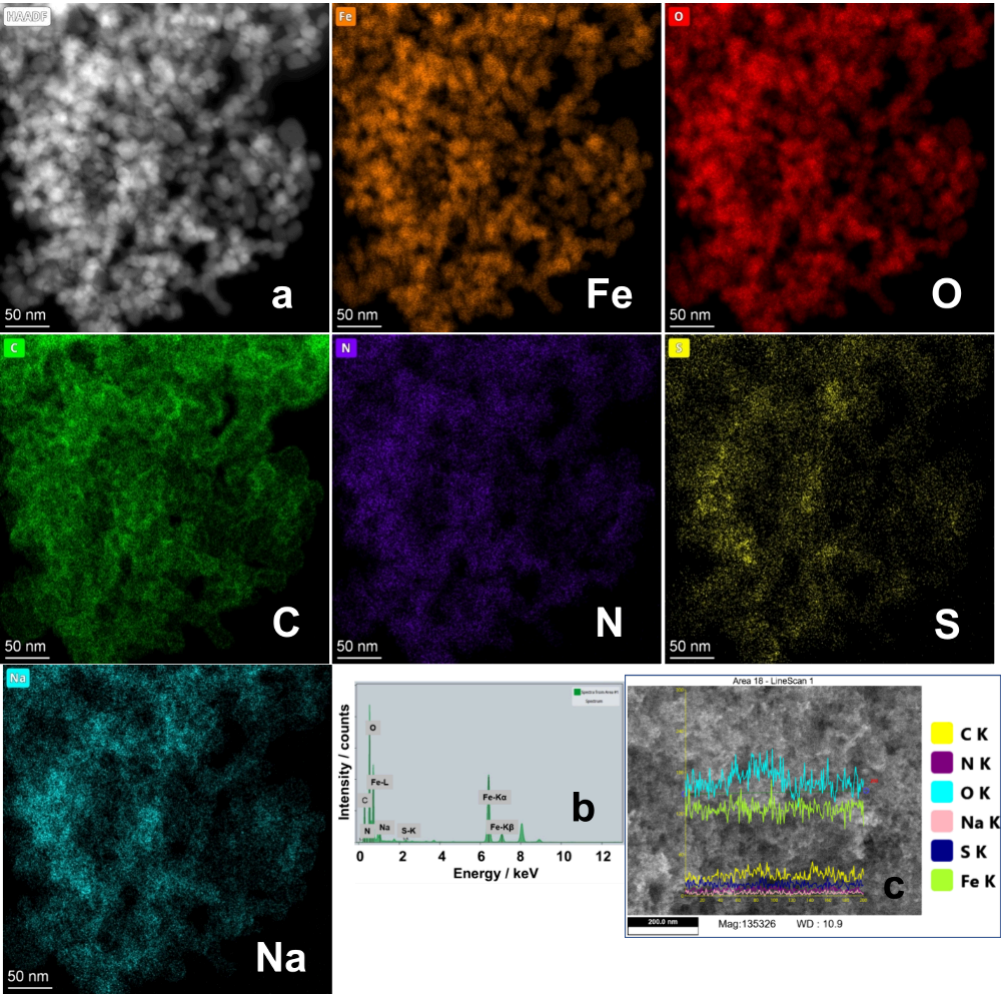
(a) HAADF TEM images of Black T-loaded β-CD-functionalized iron-oxide NPs. Frames Fe, O, C, N, S, and Na represent the elemental mapping. (b) EDS spectrum of various elements. (c) Line analysis of FESEM image of the same sample.

Figure 9 shows the TEM and EDS analysis of Black T-loaded β-CD-functionalized magnetic NPs where extraction is performed at pH 2.5. The shape and size of CD-functionalized magnetic NPs (Figure 9a) are quite similar to that of 16–6–16-functionalized magnetic NPs as noted above. The Fe and O mappings represent the iron oxide composition, whereas C mapping collectively originates from the sugar moieties of CD and Black T (EDS spectrum, Figure 9b). Here too, C is quite prominently evident from the surface coating on magnetic NPs. Both N and S mapping is only due to the extracted Black T because these elements are absent in CD-functionalized magnetic NPs. Na mapping is the consequence of the Na^+^ ions of Black T. FESEM line analysis (Figure 9c) covering a much larger area obtained from the FESEM analysis (Figure S11) further confirms the TEM analysis.

### Elemental Composition

The elemental compositions of different elements from XPS, TEM, and FESEM are compared in Table 3. Although UV–visible study demonstrates the least extraction of Red 40 and Blue 1 by surfactant- functionalized magnetic NPs, surface analysis clearly depicts their adsorption on magnetic NPs. The amount of C for P Red seems to be greater than that of other dyes-loaded 16–6–16-functionalized magnetic NPs. Similarly, the amount of O for dye-loaded CD-functionalized magnetic NPs at a fixed acidic/basic pH is higher than the samples used at neutral pH. In addition, the amount of N is mostly higher than that of S. Though this is expected for 16–6–16-functionalized magnetic NP samples because of the presence of dimeric tetraalkylammonium head groups, the higher amount of N for dye-loaded CD-functionalized magnetic NPs is obviously due to facilitated deposition of dye molecules on magnetic NPs under acidic or basic conditions. Thus, surface analysis supplements the results of UV–visible studies (Table S1) for the extraction of different dyes by surfactant and CD functionalized magnetic NPs.

**Table 3:** Elemental Composition^a^ of Different Elements in Atom % from XPS, TEM, and FESEM Analyses for Dye-Loaded Surfactant and CD Functionalized Magnetic NPs

	XPS					TEM					FESEM				
dye-loaded functionalized magnetic NPs	C	N	O	S	Fe	C	N	O	S	Fe	C	N	O	S	Fe
16–6–16–Red 40	45	3	34	1	17	51	2	29	1	16	31	2	47		19
16–6–16–Yellow 5	47	3	35	1	14	53	3	28		15	33	1	44		21
16–6–16–Yellow 6	48	2	38	1	11	54	2	25	1	18	35	3	44		17
16–6–16–Blue 1	47	1	36	1	15	49	3	27	1	20	37	2	40	1	20
16–6–16–P Red	49	2	38		12										
α-CD–CBB	28	2	44	1	25										
β-CD–CBB	28	2	43	1	26										
γ-CD–CBB	29	2	43	1	25										
β-CD–CBB (pH 2)	26	3	49	1	21										
β-CD–Black T (pH 2)	24	4	48	1	23	51	2	29	1	17	34	3	45	1	17
β-CD–C Red (pH 12)	25	2	46		26										
β-CD–P Red (pH 2)	26	3	48		23										

a The amount of atom % varies among the surface analyses of XPS, TEM, and FESEM because of the different conditions of measurements; the overall trend remains the same. Uncertainties in the measurements are less than ± 10%.

### Mechanism

**Figure 10:**
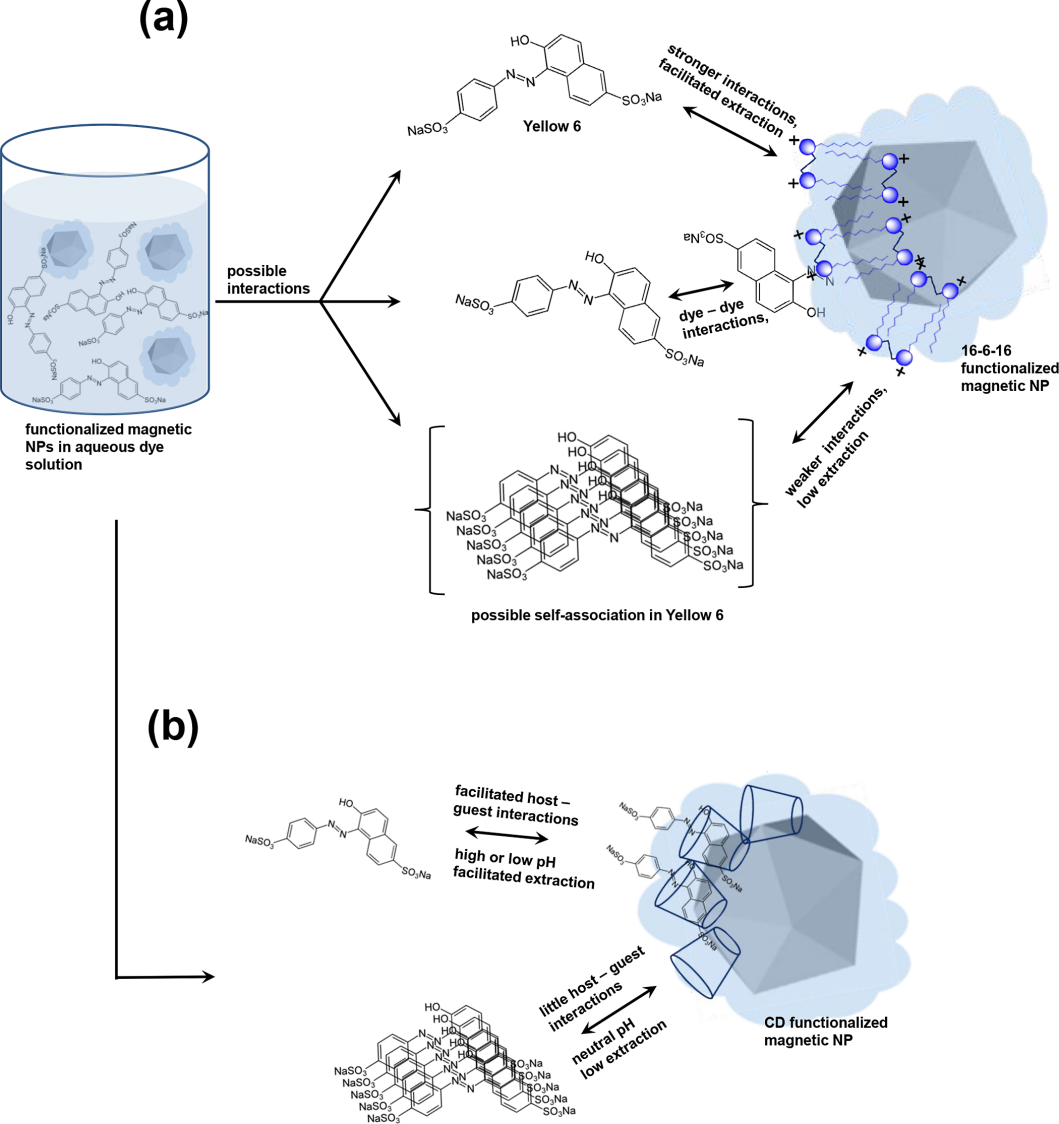
Mechanism of intermolecular interactions between dye-functionalized magnetic NPs. (a) Interactions of Yellow 6 with 16–6–16-functionalized magnetic NPs without and with self-association among the dye molecules. Double-headed arrows indicate the sufonate groups of Yellow 6 and tetraalkylammonium dimeric head groups of 16–6–16 constituting a bilayer on the surface of magnetic NPs involved in the electrostatic interactions responsible for the extraction. In the self-associated state of Yellow 6, such interactions diminish and impede the extraction. (b) Similar interactions between unassociated and associated states of Yellow 6 with CD-functionalized magnetic NPs. Extraction is fruitful when carried out at high or low pH but diminishes at neutral pH because of self-association among dye molecules.

The extraction of different dyes by water-soluble functionalized magnetic NPs^45−47^ is governed by the intermolecular interactions between the aqueous bulk-solubilized dye and the surface-immobilized surfactant or CD on magnetic NPs (Figure 10). However, the self-association among the dye molecules eventually governs the extraction.^48,49^ When self-association is low, the extraction is high, and vice versa. The self-association can happen in aqueous bulk as well as at the solid–liquid interface and depends on the molecular structure and aromatic moieties of the dye molecule.^50^ Most of the dyes used in the present study are azo dyes except P Red which possesses several aromatic moieties. A greater number of aromatic moieties add to van der Waals-like attractive forces between the dye molecules that lead to their self-association in aqueous bulk.^51^ H- and J-aggregates^52^ thus created in aqueous bulk lose their electrostatic potential to interact with the surfactant-functionalized magnetic NPs and hence impede the extraction (Figure 10a). This is quite evident in Red 40 and Blue 1 (Figure 2). Yellow 5 and 6 also show this behavior but to a much smaller extent. P Red with the lowest formula mass among all dyes studied show least self-association, with the result, its extraction is maximum (Table S1).

The self-association also reduces the ability of aromatic moieties of the dye molecule to undergo host–guest interactions with the CD cavity.^53^ Such association becomes even more arduous when dye molecules face CD immobilized on the surface of magnetic NPs (Figure 10b). The surface-immobilized CD loses the required degree of freedom for compatible host–guest complexation and hence diminishes the extraction.^25^ That is why the extraction of dyes is minimum in neutral water, where self-association among dye molecules is maximum. It becomes highly facilitated when carried out under highly acidic and/or basic pH. It ionizes the dye molecule and provides necessary surface activity which drives its solid–liquid interfacial adsorption on magnetic NPs. At the interface, the aromatic moieties of dye molecule find the CD cavity an ideal host for the host–guest interactions that provide the reason for anchoring and extraction.

## Conclusions

The results present a unique protocol for the extraction of harmful dyes from the aqueous bulk by using surfactant and CD functionalized magnetic NPs at room temperature. Extraction is dependent on the degree of self-association among the dye molecules. When the self-association is high, extraction is low, and vice versa. Among the surfactant-functionalized magnetic NPs, the polarity of the surfactant governs the extraction. Highly polar surfactants such as 16–6–16 and TriCAT possess much greater potential for extraction of dye molecules in comparison to CTAB or zwitterionic HPS.

Similarly, CD-functionalized magnetic NPs only facilitate the extraction when aromatic moieties find their way for host–guest interactions with the CD cavity. Such interactions face steric restrictions because of the immobilization of the CD cavity on the surface of magnetic NPs as well as due to the self-association of dye molecules. The neutral medium promotes the self-association of the dye and, hence, reduces the extraction by CD-functionalized magnetic NPs. However, high and low pH promotes the ionic character of dye molecules, thereby driving their solid–liquid interfacial adsorption. At the interface, dye molecules undergo host–guest interactions with the surface-immobilized CD cavity and hence facilitate the extraction. Thus, extraction of dyes depends on both the self-association of the dye as well as the surface functionalities of the functionalized magnetic NPs. A lower degree of self-association drives stronger intermolecular interactions with surface-immobilized functionalities for a meaningful extraction.

Use of functionalized iron-oxide NPs as vehicles for dye extraction is considered to be an environmentally friendly protocol because crystalline iron-oxide NPs are not expected to decompose and, hence, do not pose any environmental risk of contamination.

## Supporting Information Available

Molecular structures; sample photos; high-resolution XPS spectra; UV–visible spectra; TEM and FESEM images; HAADF TEM images; and data tables (PDF)
