# Supplementary material for: Dye Extraction by Functionalized Magnetic Nanoparticles with Surfactants and Cyclodextrins through Specific and Host–Guest Interactions
Source: Langmuir. 2025 Mar 22;41(12):8214–27. doi: 10.1021/acs.langmuir.4c05340 (PMC11966773; doi:10.1021/acs.langmuir.4c05340)
Supplement: Supplementary file 1 — la4c05340_si_001.pdf [file la4c05340_si_001.pdf]

<u>Supporting Information</u>

Dye Extraction by Functionalized Magnetic Nanoparticles with Surfactants and Cyclodextrins through Specific and Host – Guest Interactions

Manoj Kumar Goshist, Rajpreet Kaur*, Mandeep Singh Bakshi*

Department of Chemistry, Natural and Applied Sciences, University of Wisconsin - Green Bay, 2420 Nicolet Drive, Green Bay, WI 54311-7001, USA.

Corresponding author's email: *kaurr@uwgb.edu* ; *bakshim@uwgb.edu*

Number of pages 32
Figures 11
Tables 2

Fig S1a

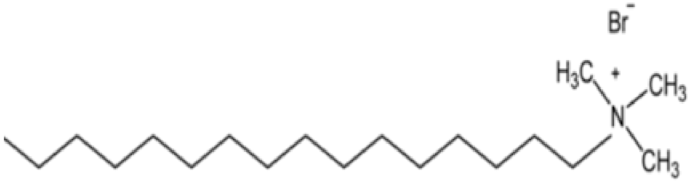

CTAB

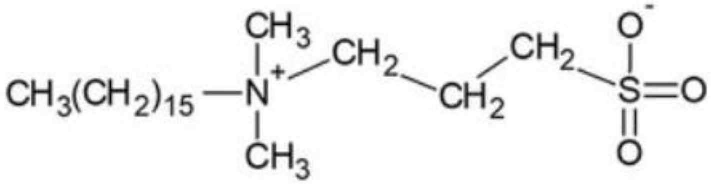

HPS

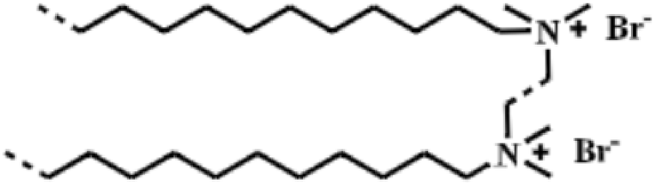

N,N'-Dialkyl-N,N,N'N'-tetramethylhexanediyldiammonium bromide (16-6-16)

Alkyl = hexadecyl (16)

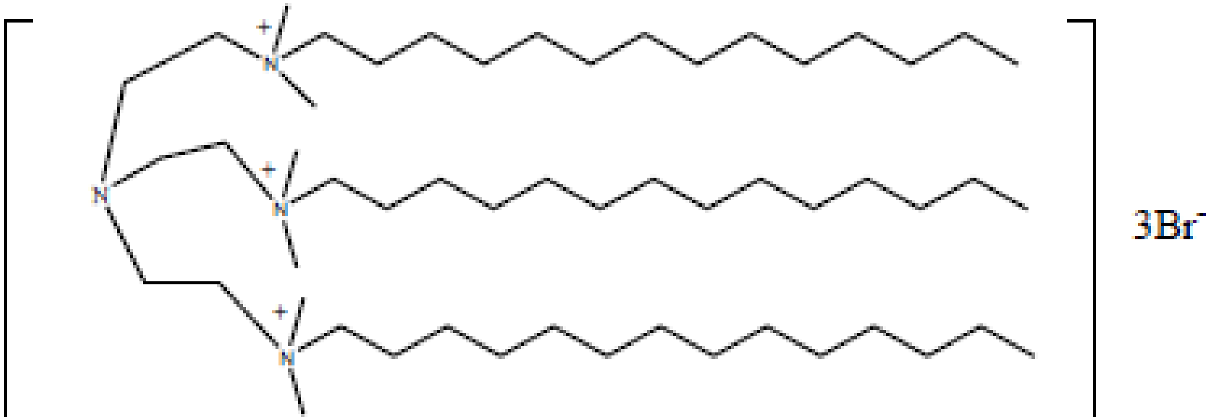

tris(2-(N-dodecyl N,N-dimethylammonio)ethylamine) tribromide (TriCAT)

Fig S1a. Molecular structures of CTAB, HPS, 16-6-16, and TriCAT.

Fig S1b

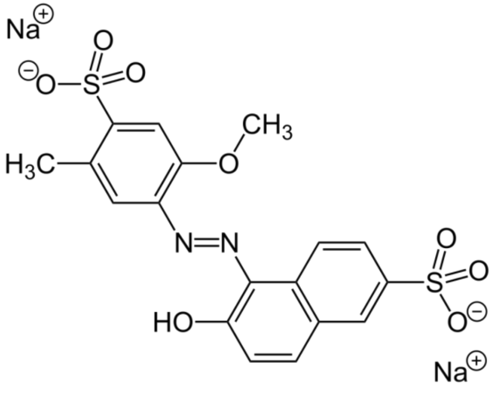

Red 40 (492.4 g/mol)

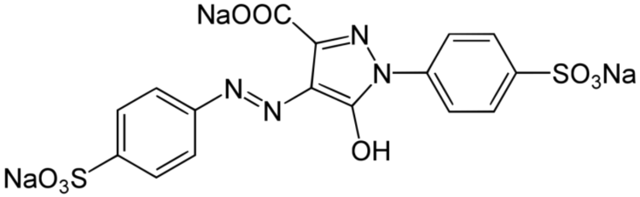

Yellow 5 (534.3 g/mol)

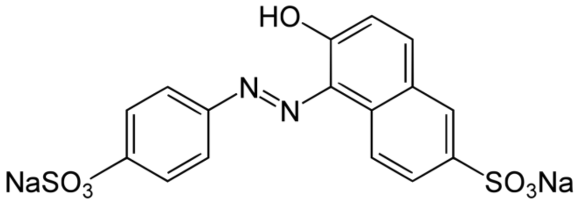

Yellow 6 (452.4 g/mol)

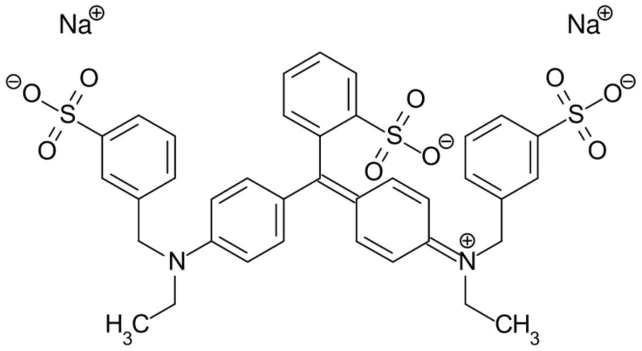

Blue 1 (792.8 g/mol)

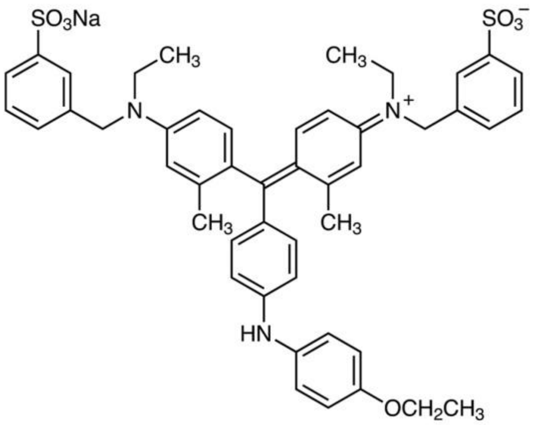

Coomassie Brilliant Blue G-250

(CBB, 826 g/mol)

Fig S1b. Molecular structures of Red 40, Yellow 5, Yellow 6, Blue 1, and CBB.

Fig S1c

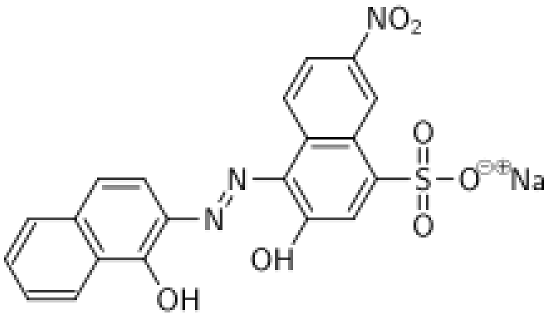

Eriochrome Black T (Black T)
461.4 g/mol

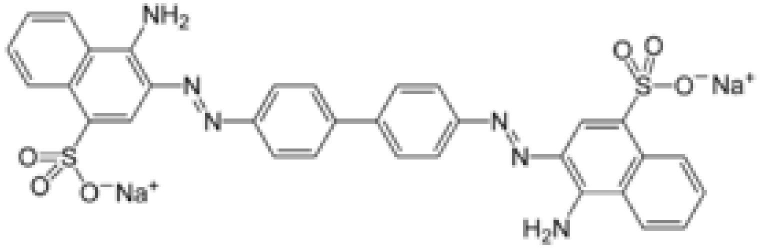

Congo red (C Red)
696.7 g/mol

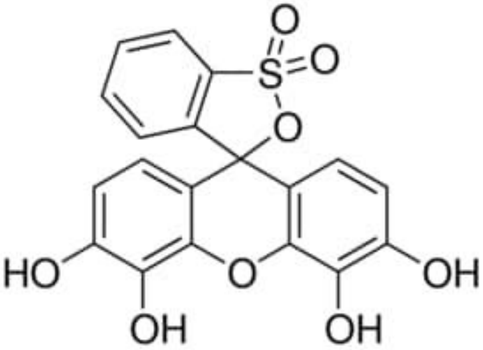

Pyrogallol Red (P Red)
400.4 g/mol

Fig S1c. Molecular structures of Black T, C Red, and P Red.

Fig S1d

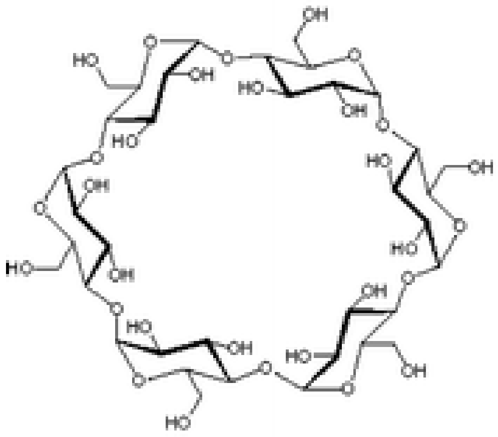

α-cyclodextrin

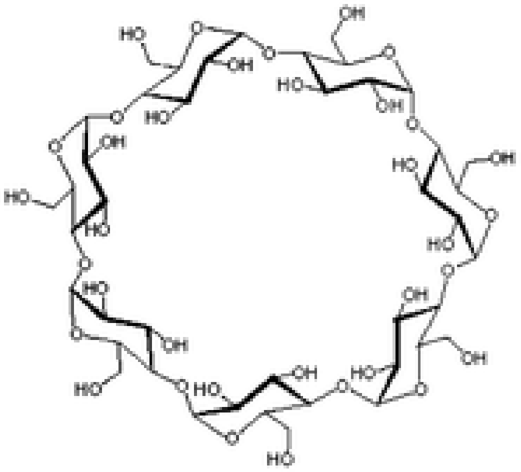

β-cyclodextrin

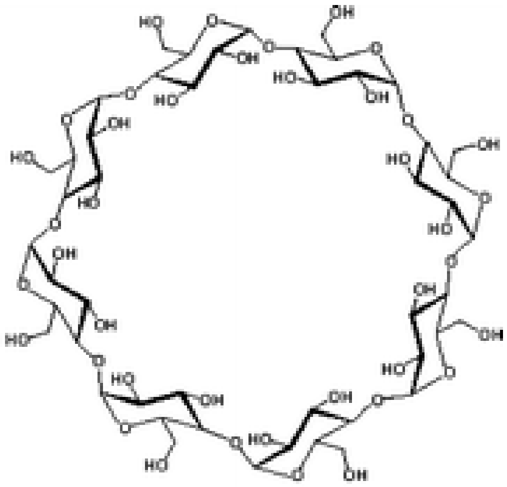

γ-cyclodextrin

Fig S1d. Molecular structures of different cyclodextrin molecules.

Fig S2a

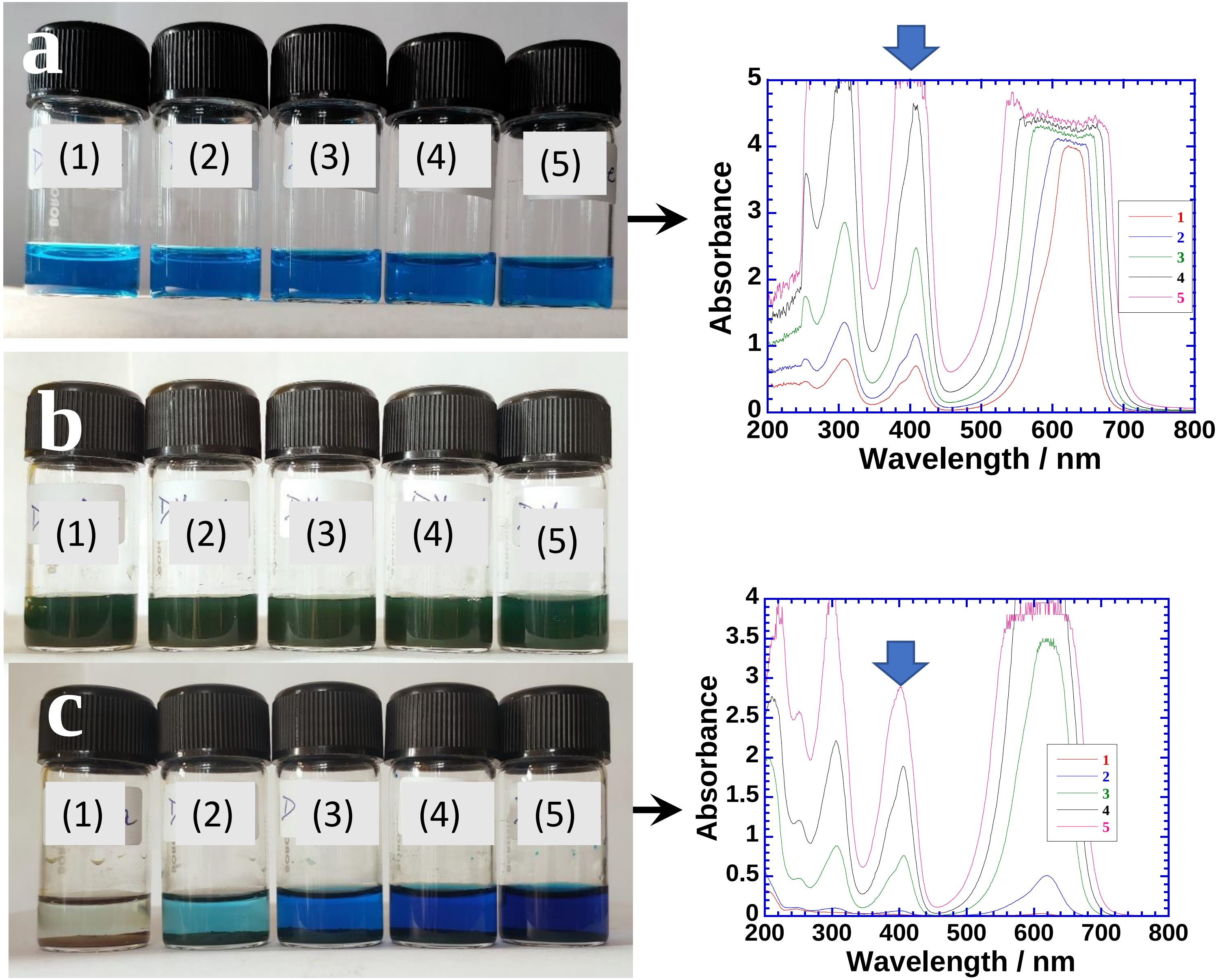

Fig S2a. (a) Photos of aqueous Blue 1 solutions of (1) 0.05, (2) 0.1, (3) 0.2, (4) 0.4, and (5) 0.8 mM. (b) Photos after the addition of 40 mM 16-6-16 functionalized magnetic NPs in each bottle. (c) Photos after one week. Corresponding UV-visible spectra of the solution of each sample bottle of (a) and (c).

Fig S2b

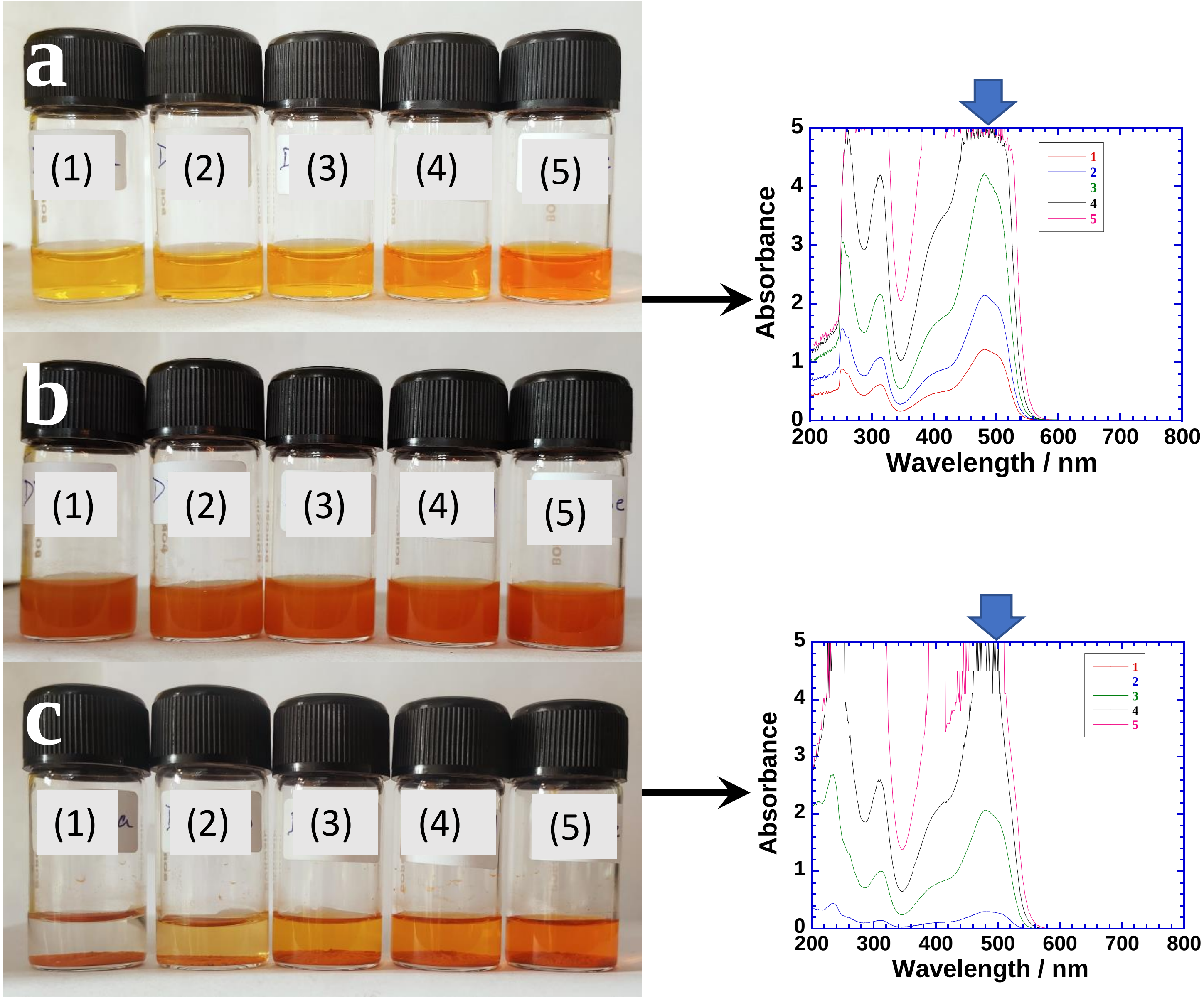

Fig S2b. (a) Photos of aqueous Yellow 6 solutions of (1) 0.05, (2) 0.1, (3) 0.2, (4) 0.4, and (5) 0.8 mM. (b) Photos after the addition of 40 mM 16-6-16 functionalized magnetic NPs in each bottle. (c) Photos after one week. Corresponding UV-visible spectra of the solution of each sample bottle of (a) and (c).

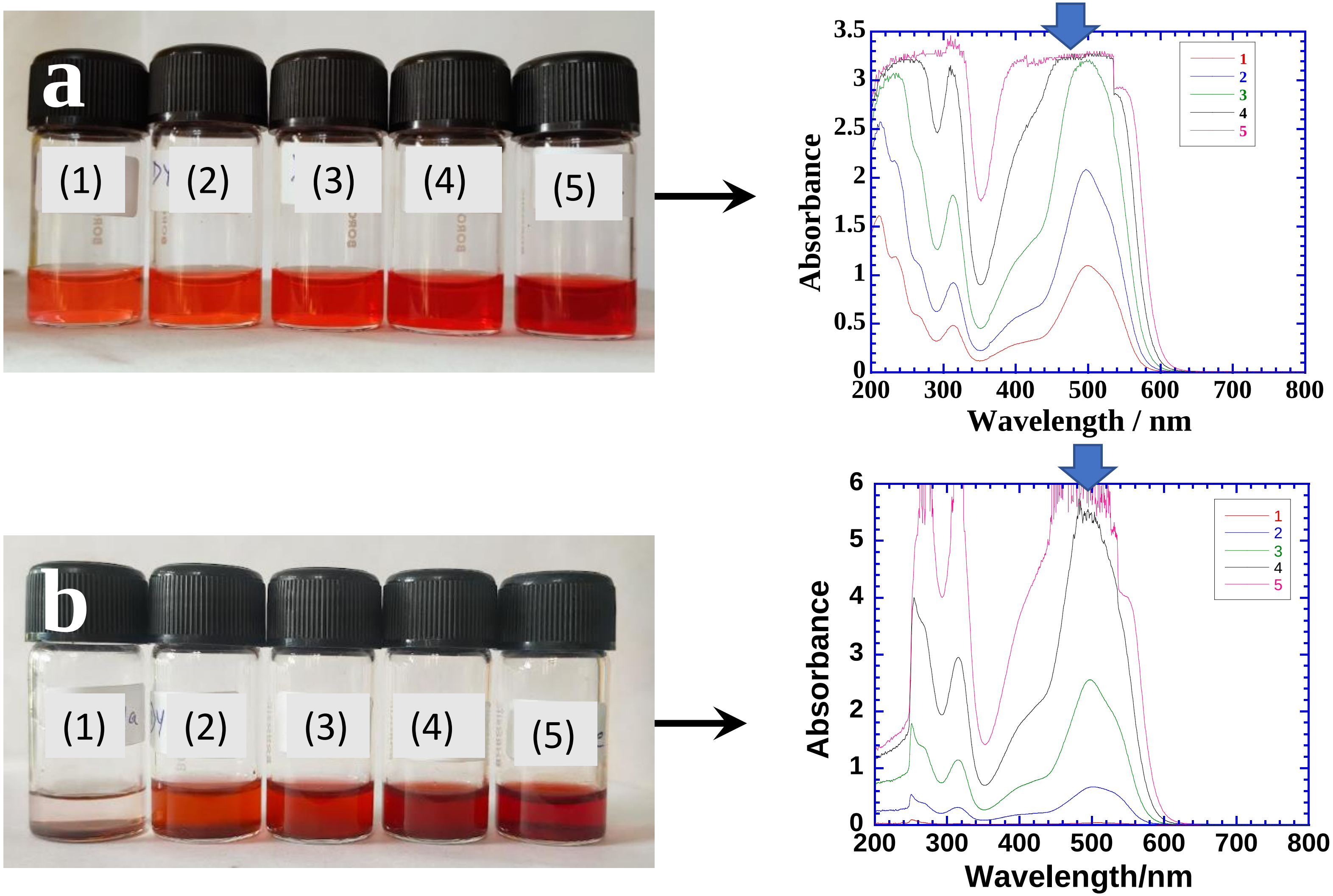

Fig S3a. (a) Photos of aqueous Red 40 solutions of (1) 0.05, (2) 0.1, (3) 0.2, (4) 0.4, and (5) 0.8 mM. (b) Photos after one week with the addition of 40 mM CTAB functionalized magnetic NPs in each bottle. Corresponding UV-visible spectra of the solution of each sample bottle of (a) and (b).

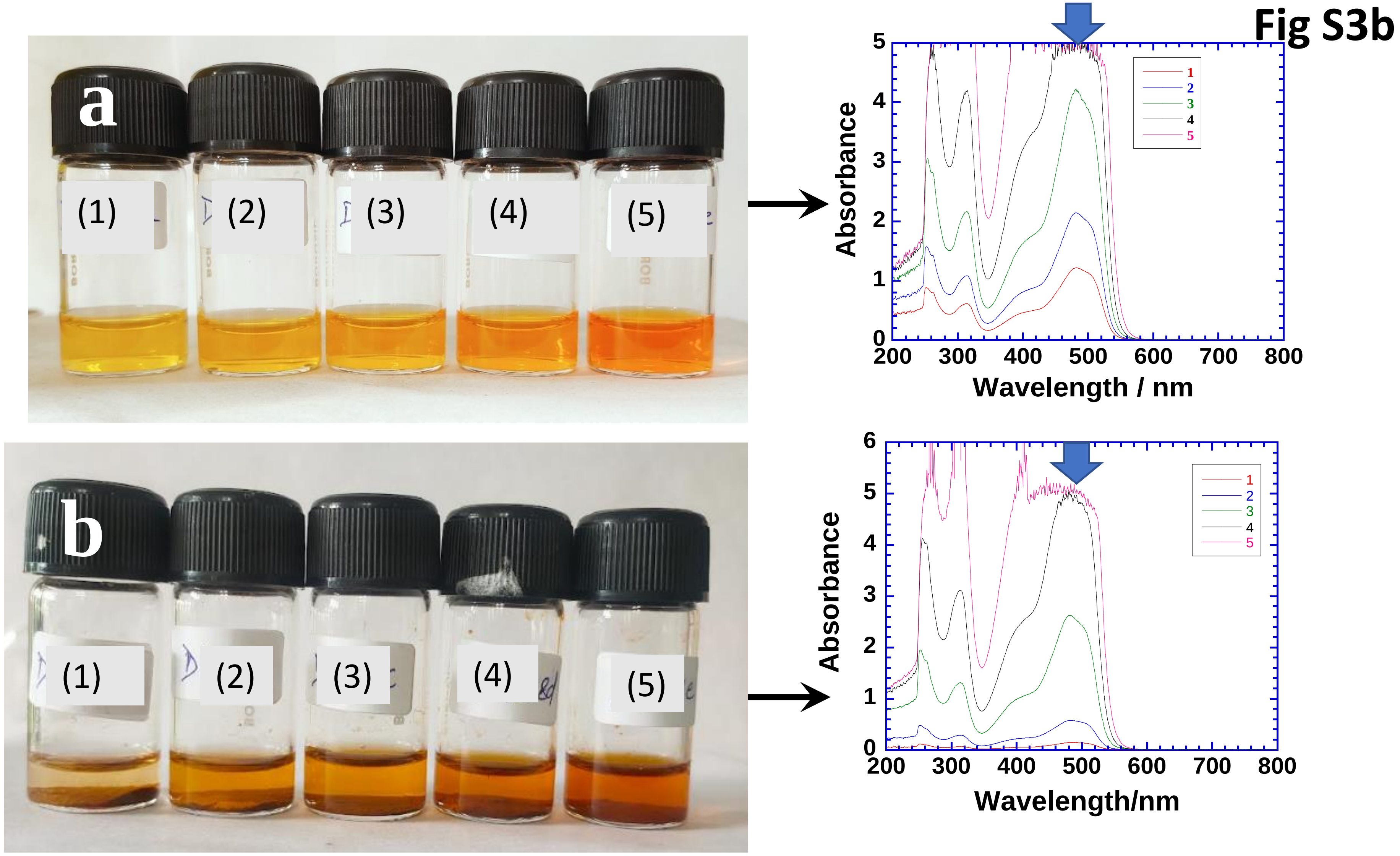

Fig S3b. (a) Photos of aqueous Yellow 6 solutions of (1) 0.05, (2) 0.1, (3) 0.2, (4) 0.4, and (5) 0.8 mM. (b) Photos after one week with the addition of 40 mM CTAB functionalized magnetic NPs in each bottle. Corresponding UV-visible spectra of the solution of each sample bottle of (a) and (b).

Fig S3c

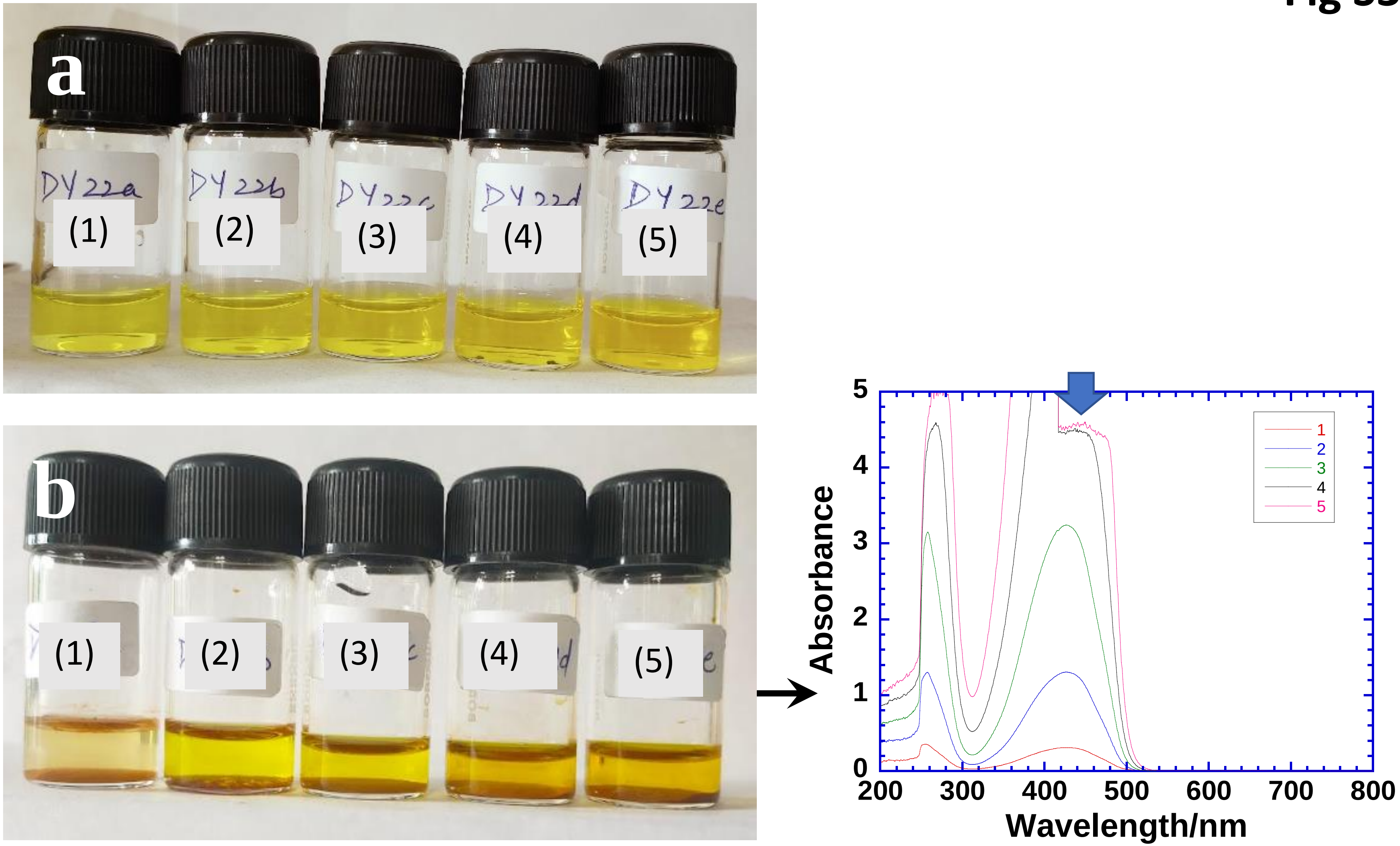

Fig S3c. (a) Photos of aqueous Yellow 5 solutions of (1) 0.05, (2) 0.1, (3) 0.2, (4) 0.4, and (5) 0.8 mM. (b) Photos after one week with the addition of 40 mM CTAB functionalized magnetic NPs in each bottle. Corresponding UV-visible spectra of the solution of each sample bottle of (b).

Fig S3d

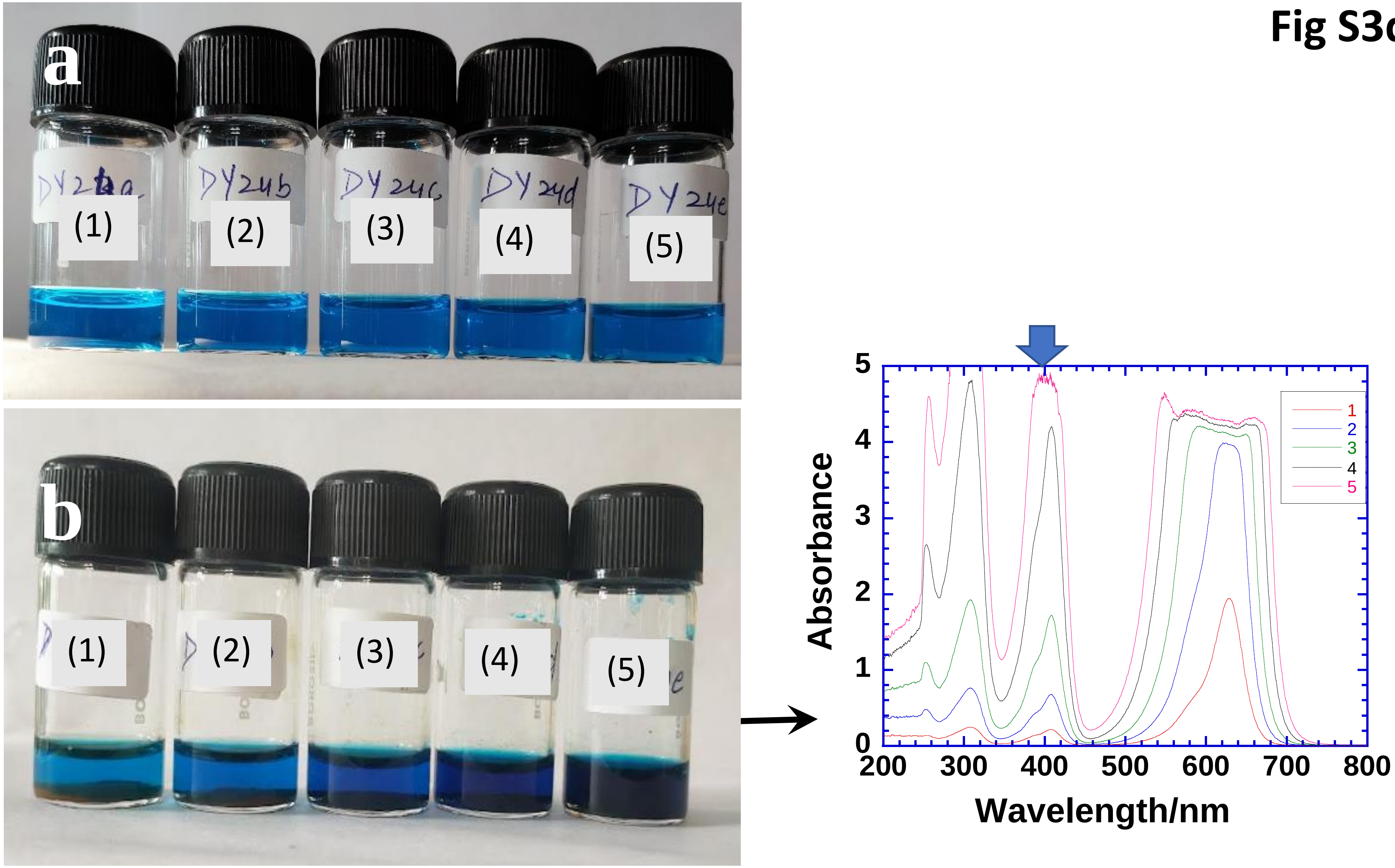

Fig S3d. (a) Photos of aqueous Blue 1 solutions of (1) 0.05, (2) 0.1, (3) 0.2, (4) 0.4, and (5) 0.8 mM. (b) Photos after one week with the addition of 40 mM CTAB functionalized magnetic NPs in each bottle. Corresponding UV-visible spectra of the solution of each sample bottle of (b).

Fig S4a

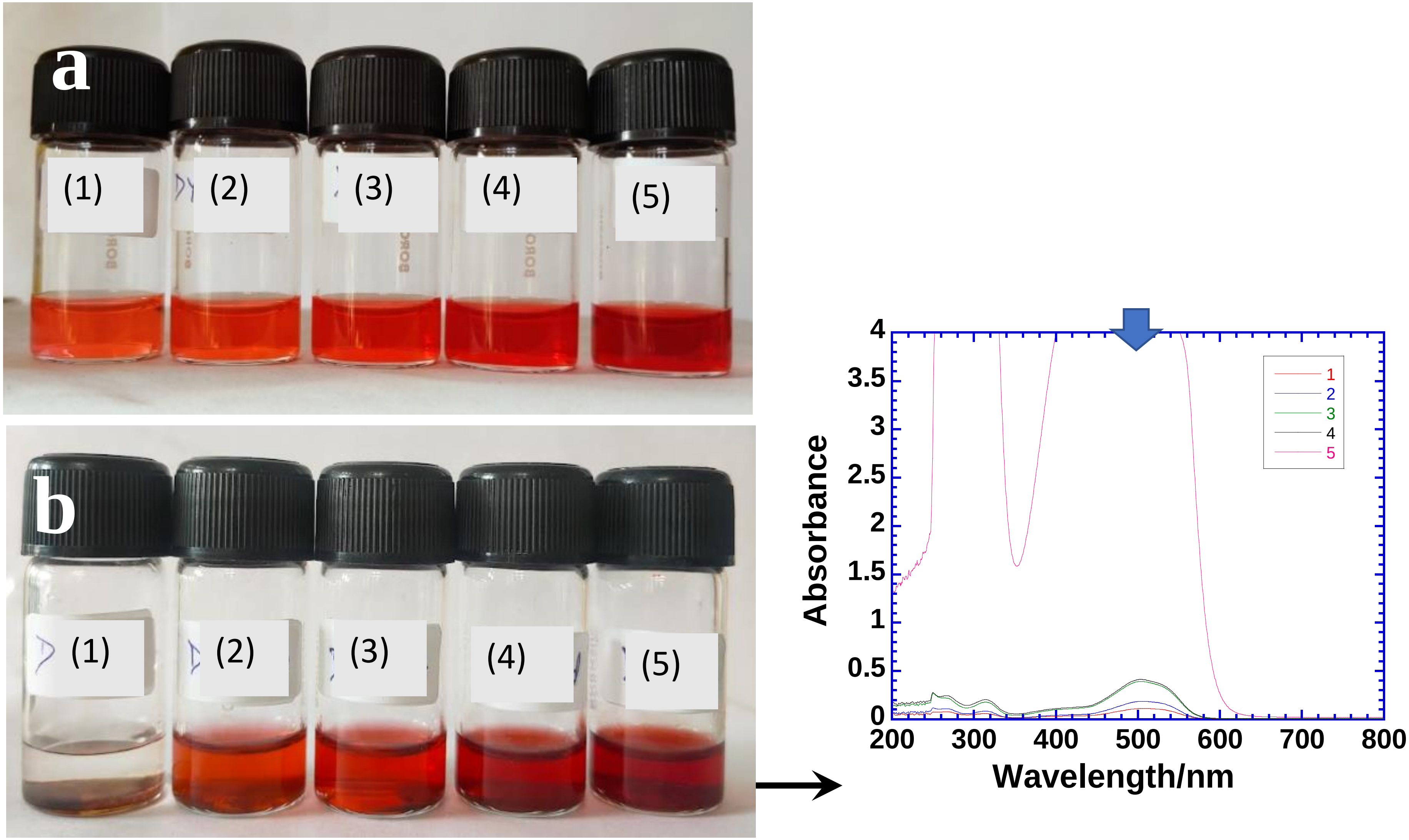

Fig S4a. (a) Photos of aqueous Red 40 solutions of (1) 0.05, (2) 0.1, (3) 0.2, (4) 0.4, and (5) 0.8 mM. (b) Photos after one week with the addition of 40 mM Tri-CAT functionalized magnetic NPs in each bottle. Corresponding UV-visible spectra of the solution of each sample bottle of (b).

Fig S4b

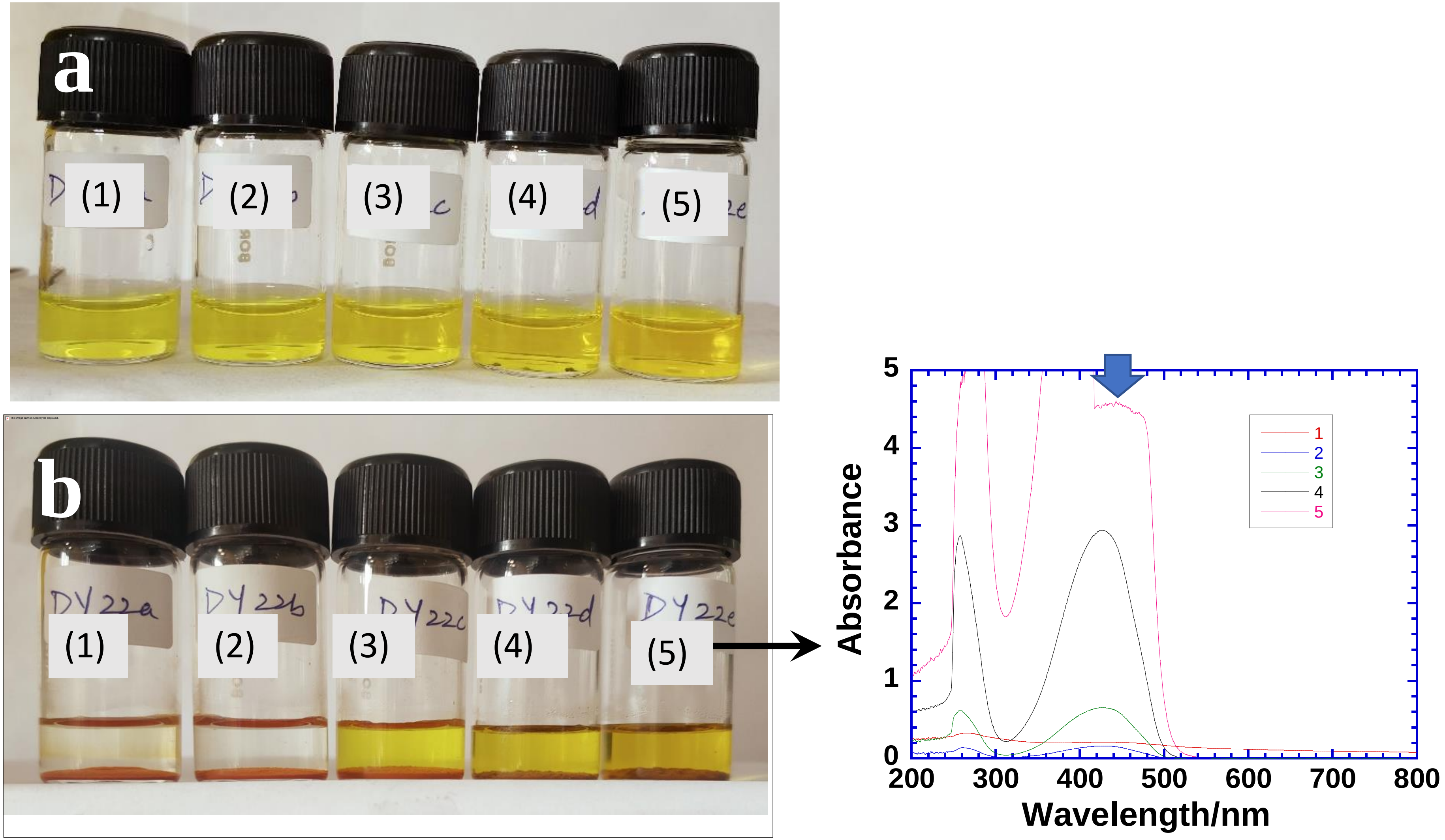

Fig S4b. (a) Photos of aqueous Yellow 5 solutions of (1) 0.05, (2) 0.1, (3) 0.2, (4) 0.4, and (5) 0.8 mM. (b) Photos after one week with the addition of 40 mM Tri-CAT functionalized magnetic NPs in each bottle. Corresponding UV-visible spectra of the solution of each sample bottle of (b).

Fig S4c

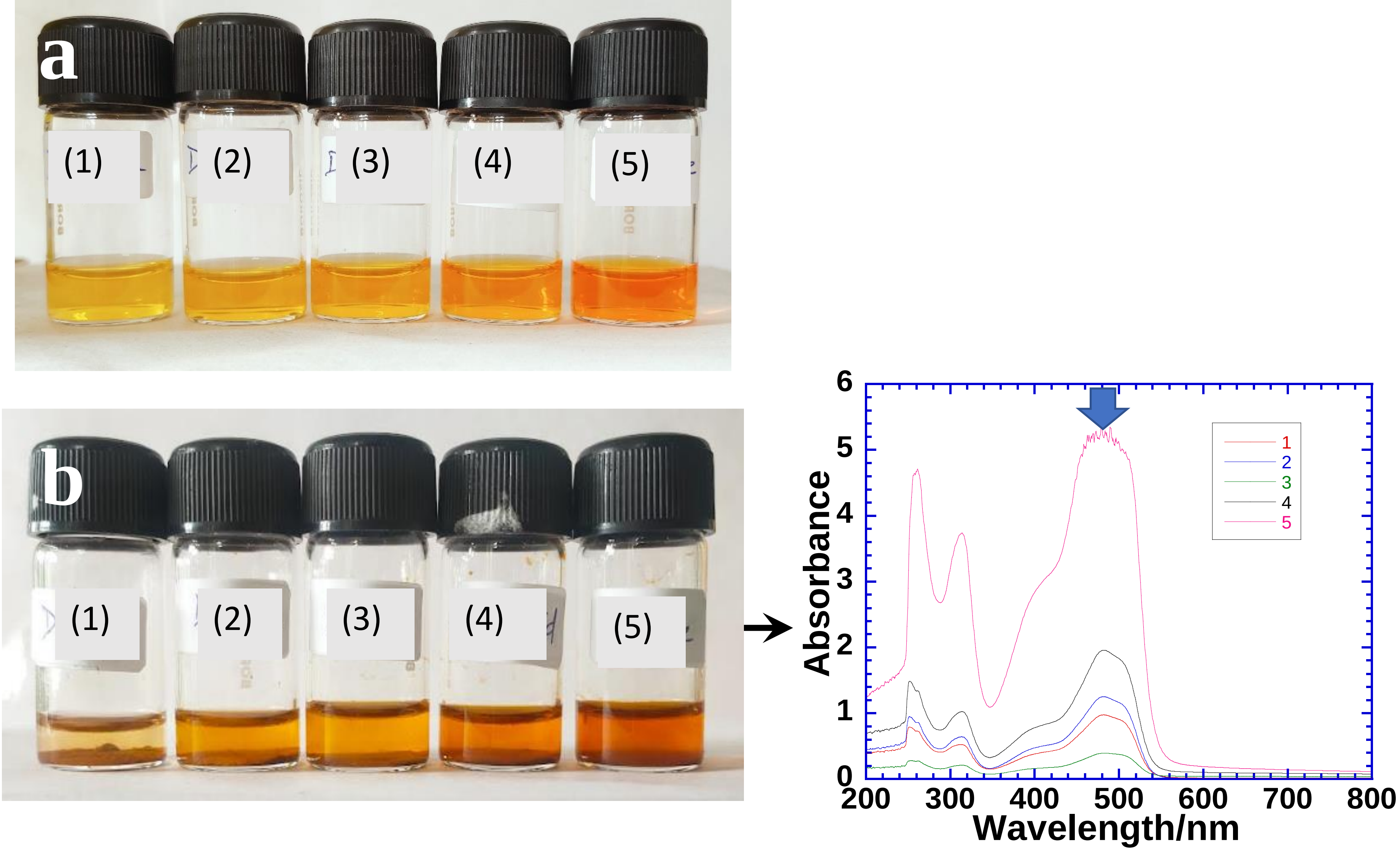

Fig S4c. (a) Photos of aqueous Yellow 6 solutions of (1) 0.05, (2) 0.1, (3) 0.2, (4) 0.4, and (5) 0.8 mM. (b) Photos after one week with the addition of 40 mM Tri-CAT functionalized magnetic NPs in each bottle. Corresponding UV-visible spectra of the solution of each sample bottle of (b).

Fig S4d

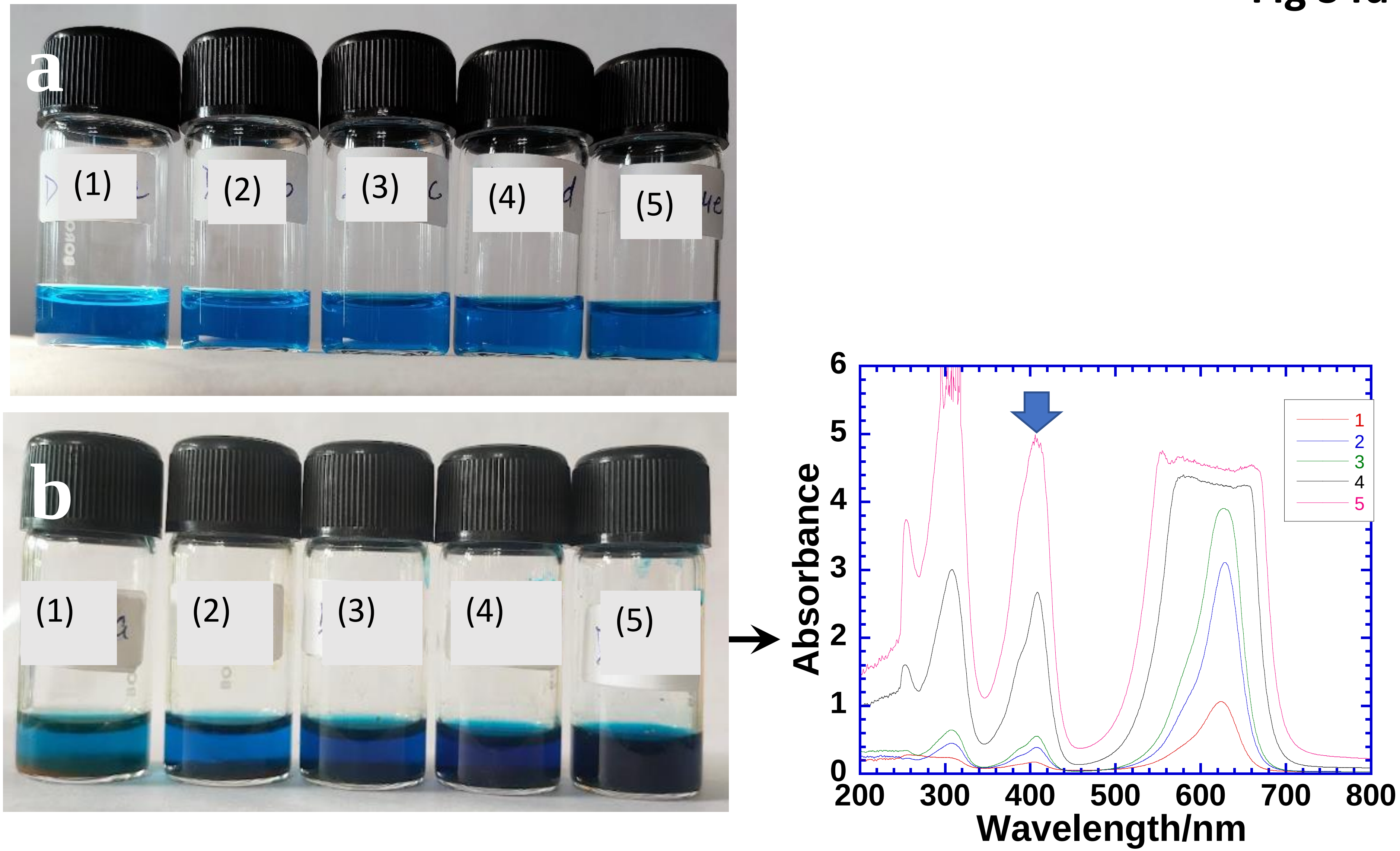

Fig S4d. (a) Photos of aqueous Blue 1 solutions of (1) 0.05, (2) 0.1, (3) 0.2, (4) 0.4, and (5) 0.8 mM. (b) Photos after one week with the addition of 40 mM Tri-CAT functionalized magnetic NPs in each bottle. Corresponding UV-visible spectra of the solution of each sample bottle of (b).

Fig S5a

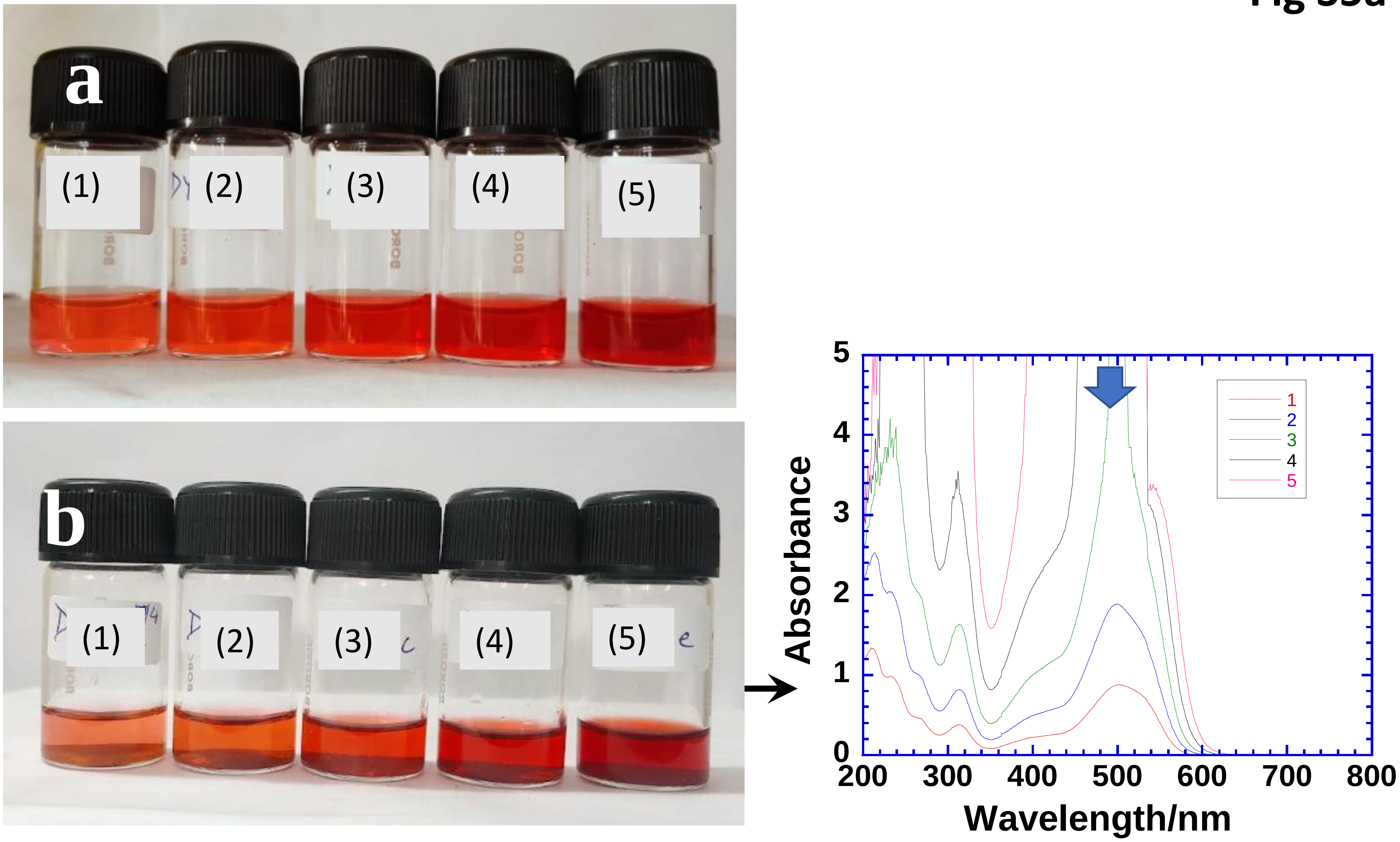

Fig S5a. (a) Photos of aqueous Red 40 solutions of (1) 0.05, (2) 0.1, (3) 0.2, (4) 0.4, and (5) 0.8 mM. (b) Photos after one week with the addition of 40 mM HPS functionalized magnetic NPs in each bottle. Corresponding UV-visible spectra of the solution of each sample bottle of (b).

Fig S5b

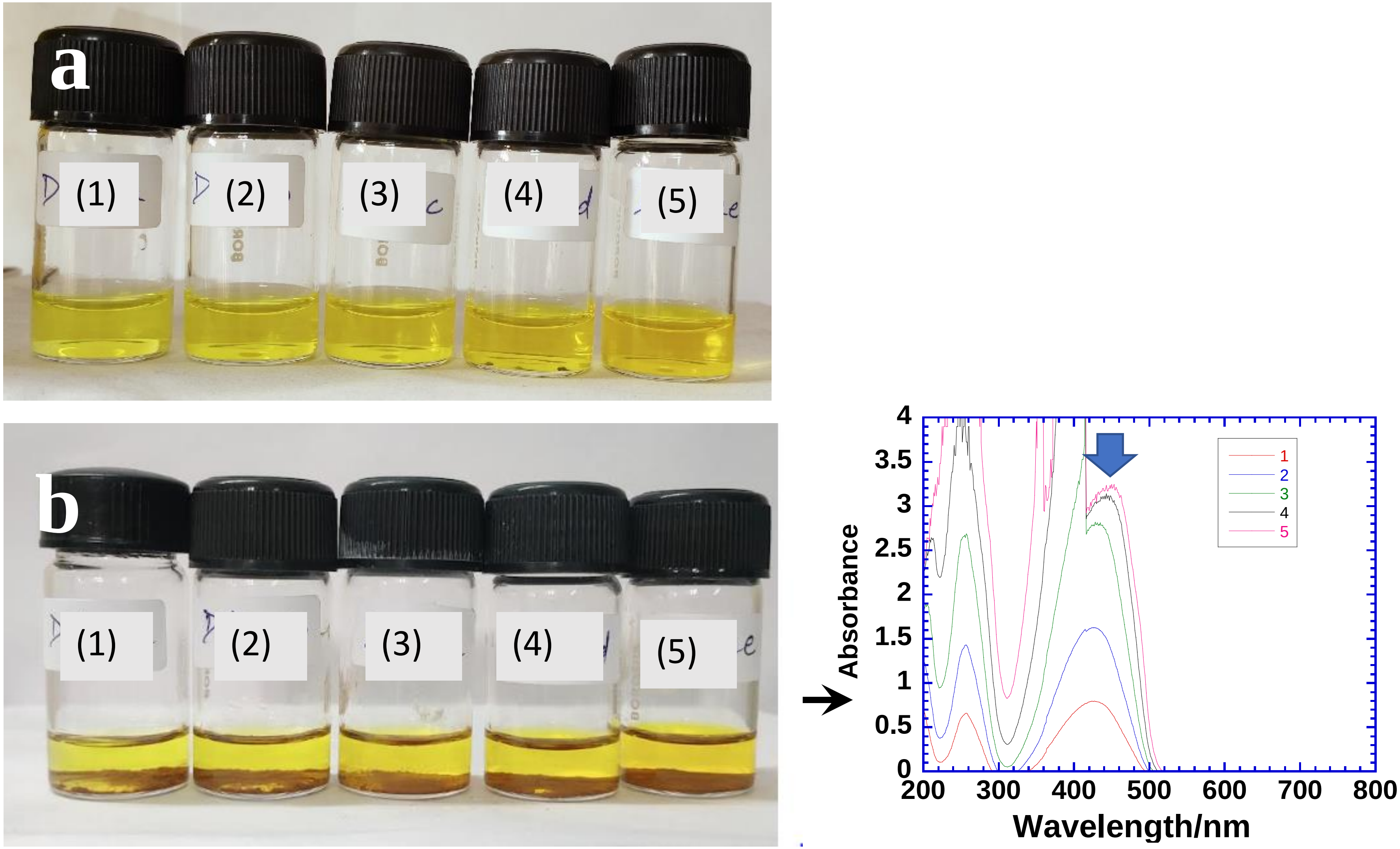

Fig S5b. (a) Photos of aqueous Yellow 5 solutions of (1) 0.05, (2) 0.1, (3) 0.2, (4) 0.4, and (5) 0.8 mM. (b) Photos after one week with the addition of 40 mM HPS functionalized magnetic NPs in each bottle. Corresponding UV-visible spectra of the solution of each sample bottle of (b).

Fig S5c

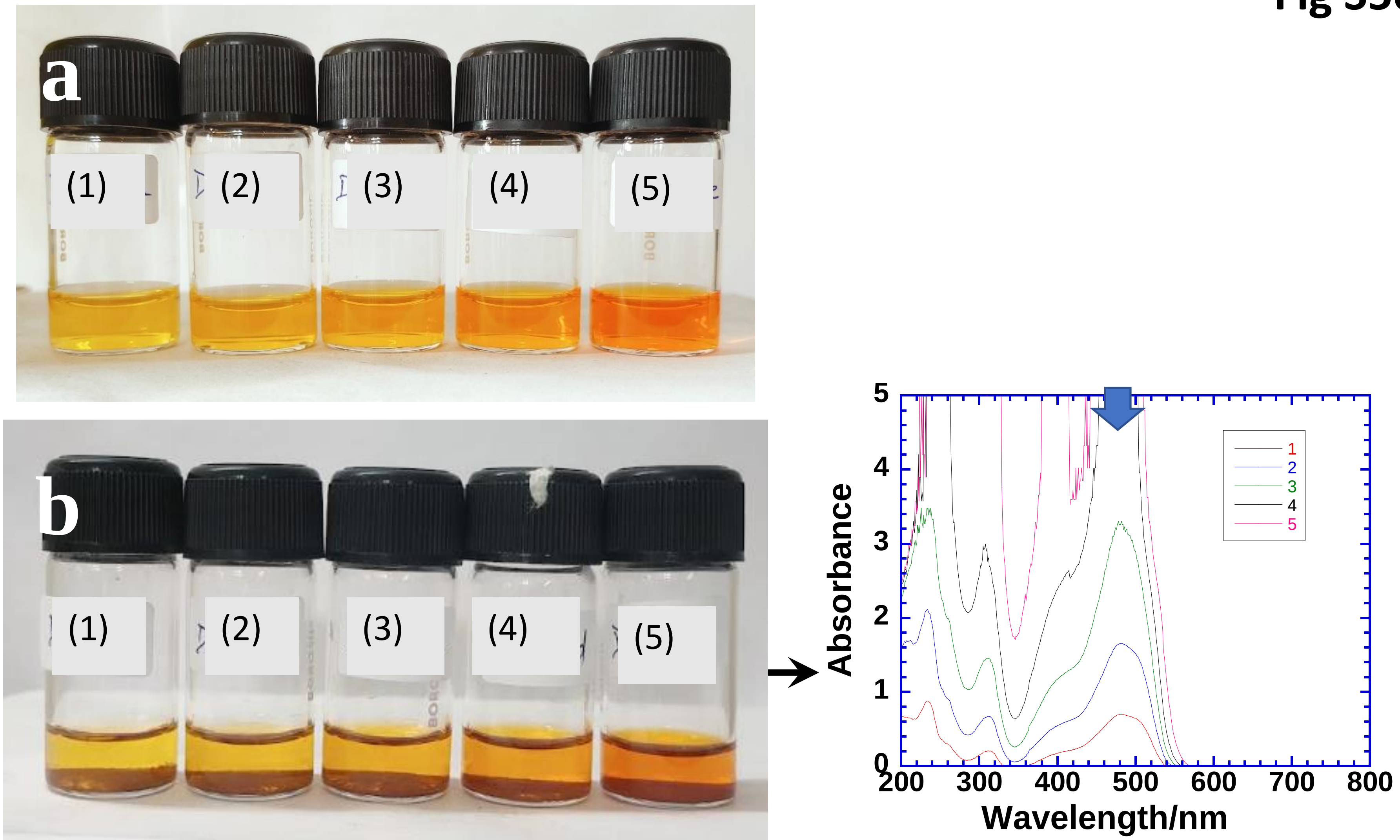

Fig S5c. (a) Photos of aqueous Yellow 6 solutions of (1) 0.05, (2) 0.1, (3) 0.2, (4) 0.4, and (5) 0.8 mM. (b) Photos after one week with the addition of 40 mM HPS functionalized magnetic NPs in each bottle. Corresponding UV-visible spectra of the solution of each sample bottle of (b).

Fig S5d

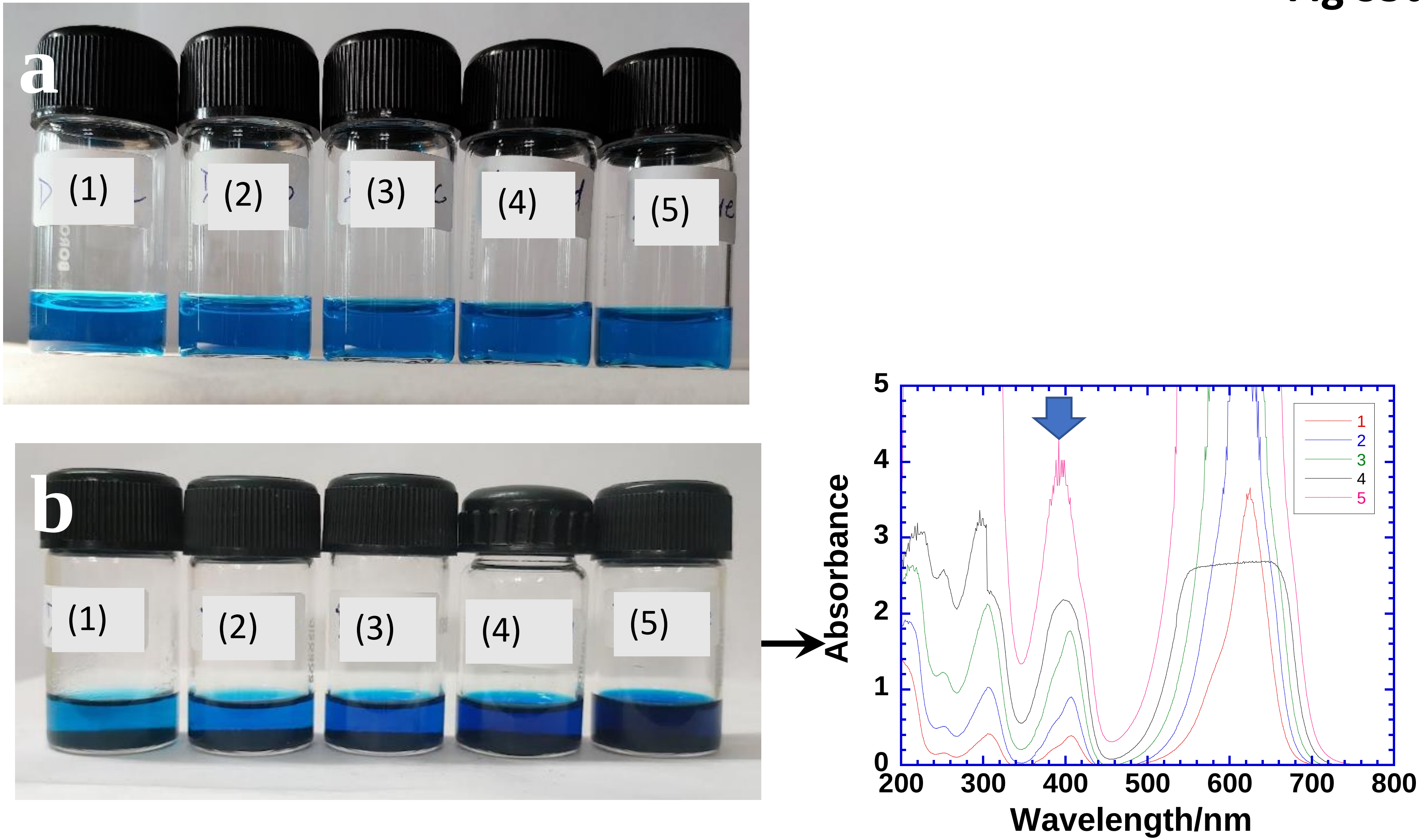

Fig S5d. (a) Photos of aqueous Blue 1 solutions of (1) 0.05, (2) 0.1, (3) 0.2, (4) 0.4, and (5) 0.8 mM. (b) Photos after one week with the addition of 40 mM HPS functionalized magnetic NPs in each bottle. Corresponding UV-visible spectra of the solution of each sample bottle of (b).

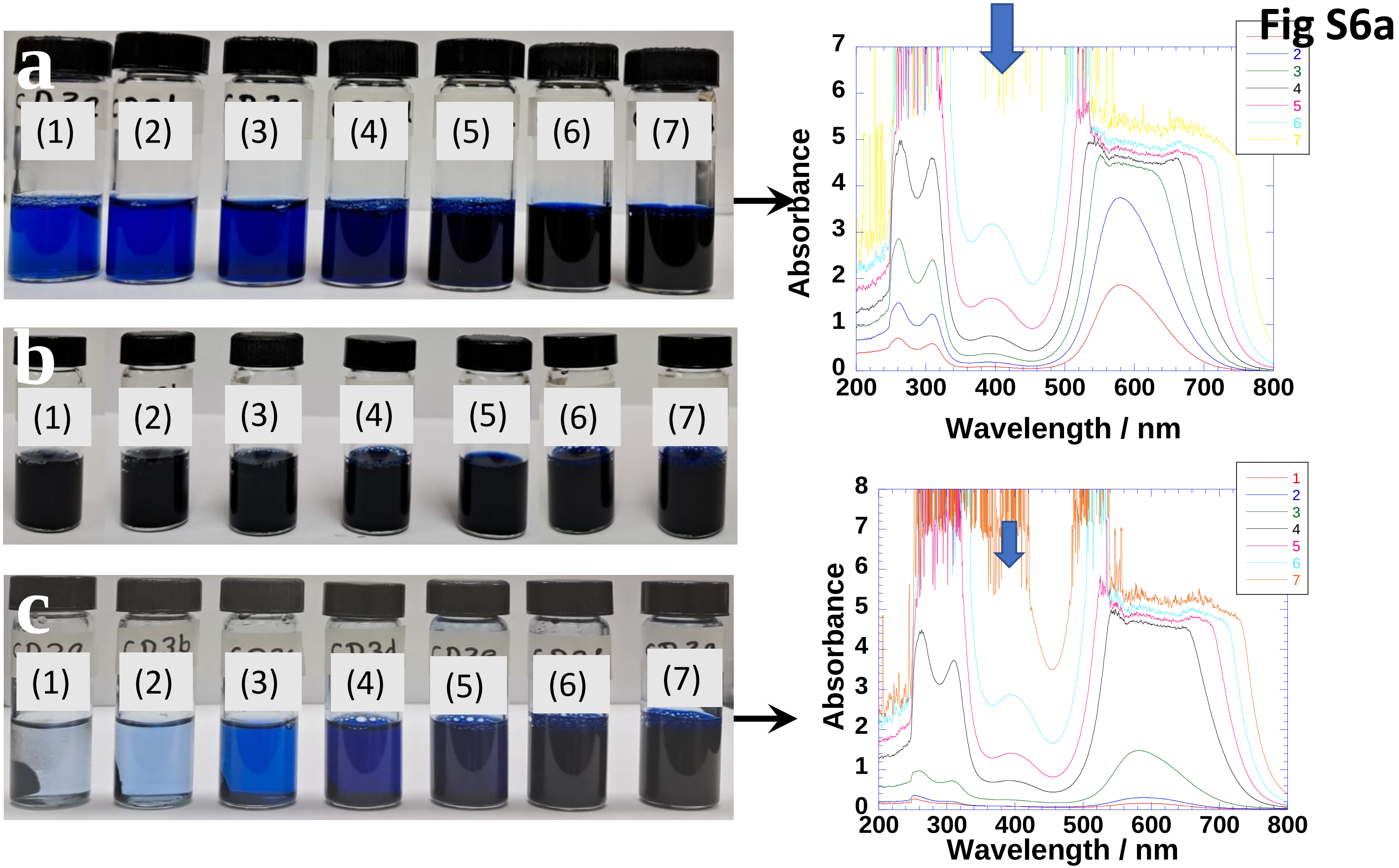

Fig S6a. (a) Photos of aqueous Coomassie Brilliant Blue solutions of (1) 0.05, (2) 0.1, (3) 0.2, (4) 0.4, (5) 0.8 mM, (6) 1.6 mM, and (7) 3.2 mM (b) Photos after addition of 5mM α-Cyclodextrin functionalized magnetic NPs in each bottle. (c) Photos after one week. Corresponding UV-visible spectra of the solution of each sample bottle of (a) and (c).

Fig S6b

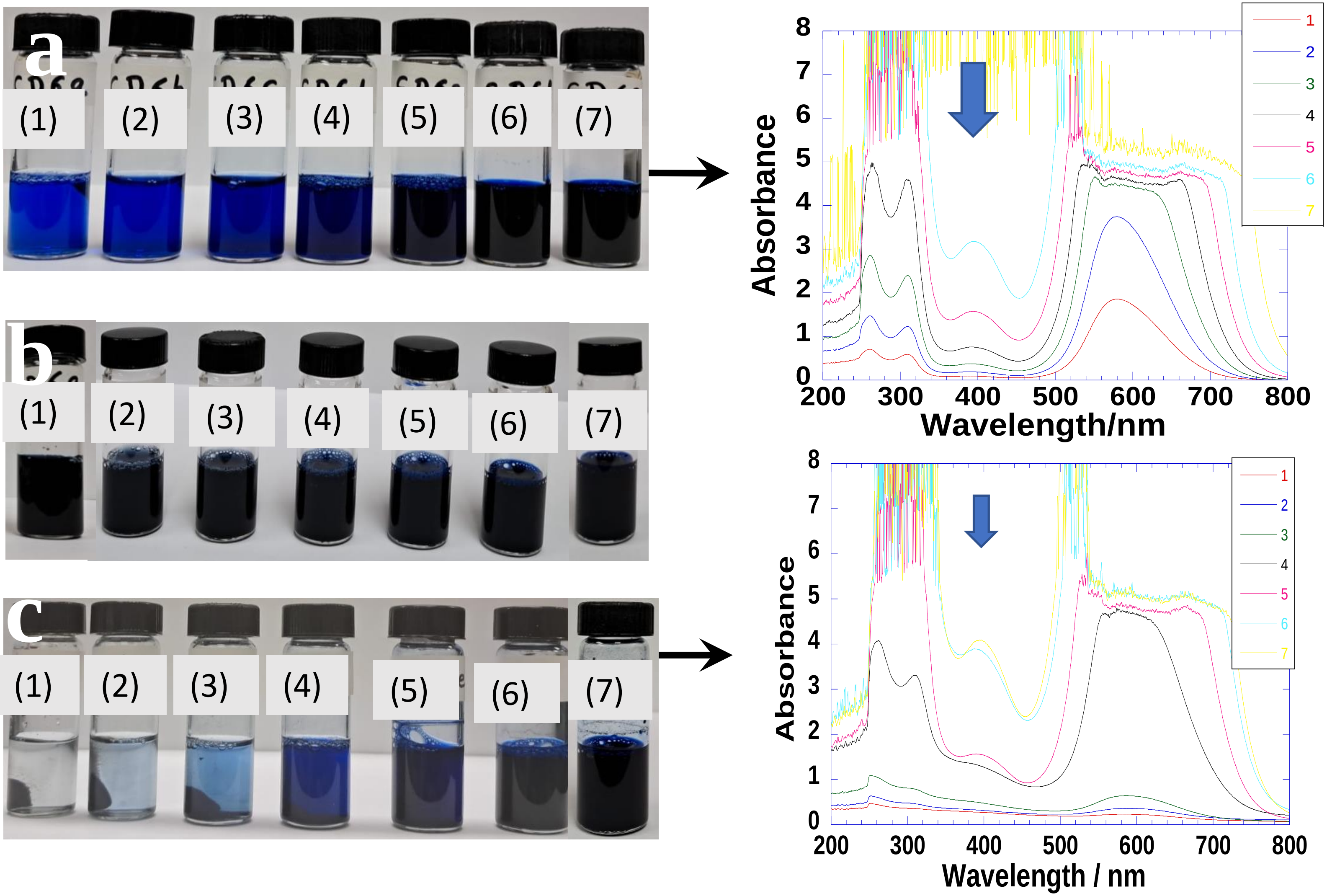

Fig S6b. (a) Photos of aqueous Coomassie Brilliant Blue solutions of (1) 0.05, (2) 0.1, (3) 0.2, (4) 0.4, and (5) 0.8 mM, (6) 1.6 mM, and (7) 3.2 mM (b) Photos after addition of 5mM β-Cyclodextrin functionalized magnetic NPs in each bottle. (c) Photos after one week Corresponding UV-visible spectra of the solution of each sample bottle of (a) and (c).

Fig S6c

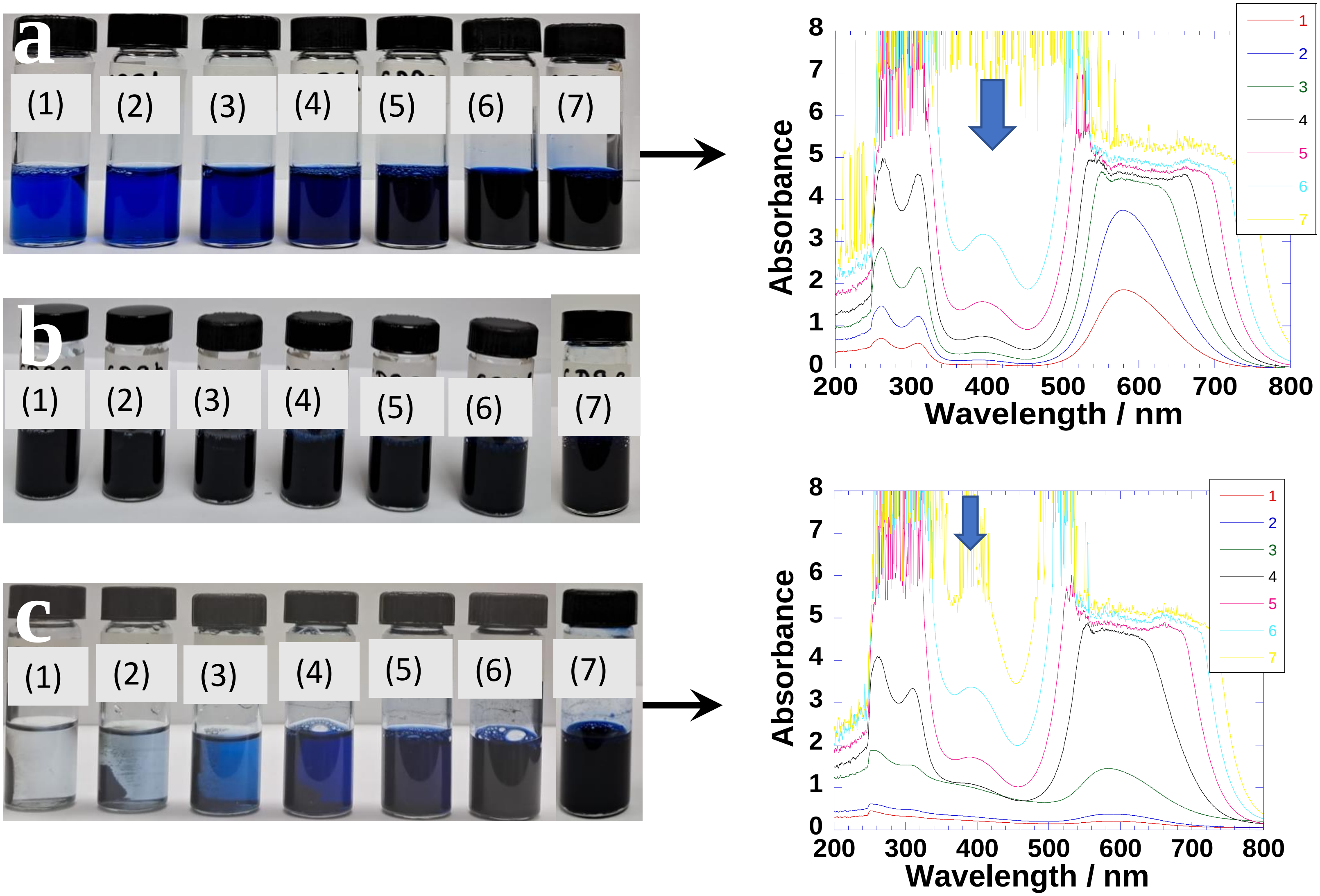

Fig S6c. (a) Photos of aqueous Coomassie Brilliant Blue solutions of (1) 0.05, (2) 0.1, (3) 0.2, (4) 0.4, (5) 0.8 mM, (6) 1.6 mM, and (7) 3.2 mM (b) Photos after addition of 5mM γ-Cyclodextrin functionalized magnetic NPs in each bottle. (c) Photos after one week Corresponding UV-visible spectra of the solution of each sample bottle of (a) and (c).

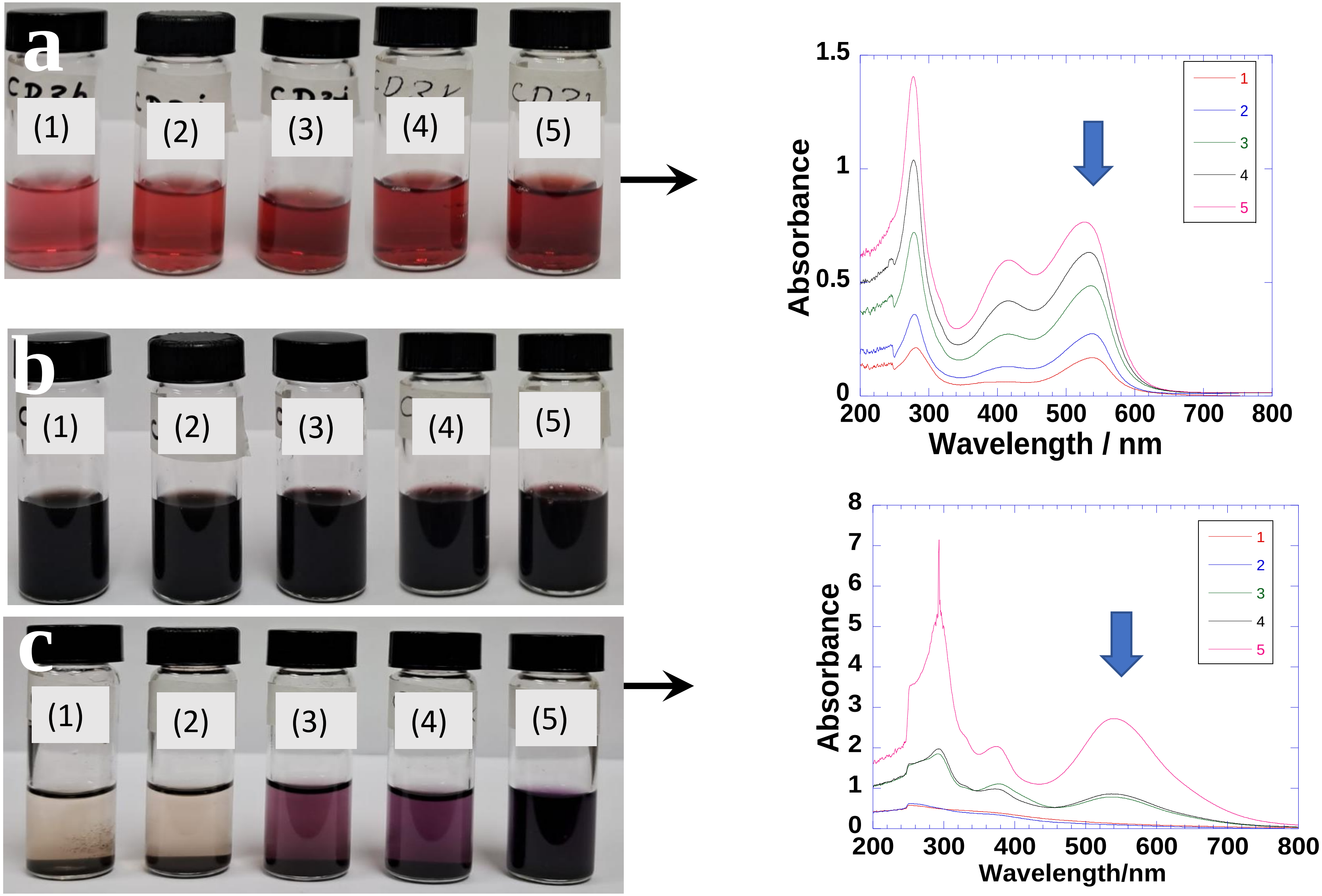

Fig S7a. (a) Photos of aqueous Pyrogallol Red solutions of (1) 0.05, (2) 0.1, (3) 0.2, (4) 0.3, and (5) 0.4 mM. (b) Photos after the addition of 5mM α-Cyclodextrin functionalized magnetic NPs in each bottle. (c) Photo after one week. Corresponding UV-visible spectra of the solution of each sample bottle of (a) and (c).

Fig S7b

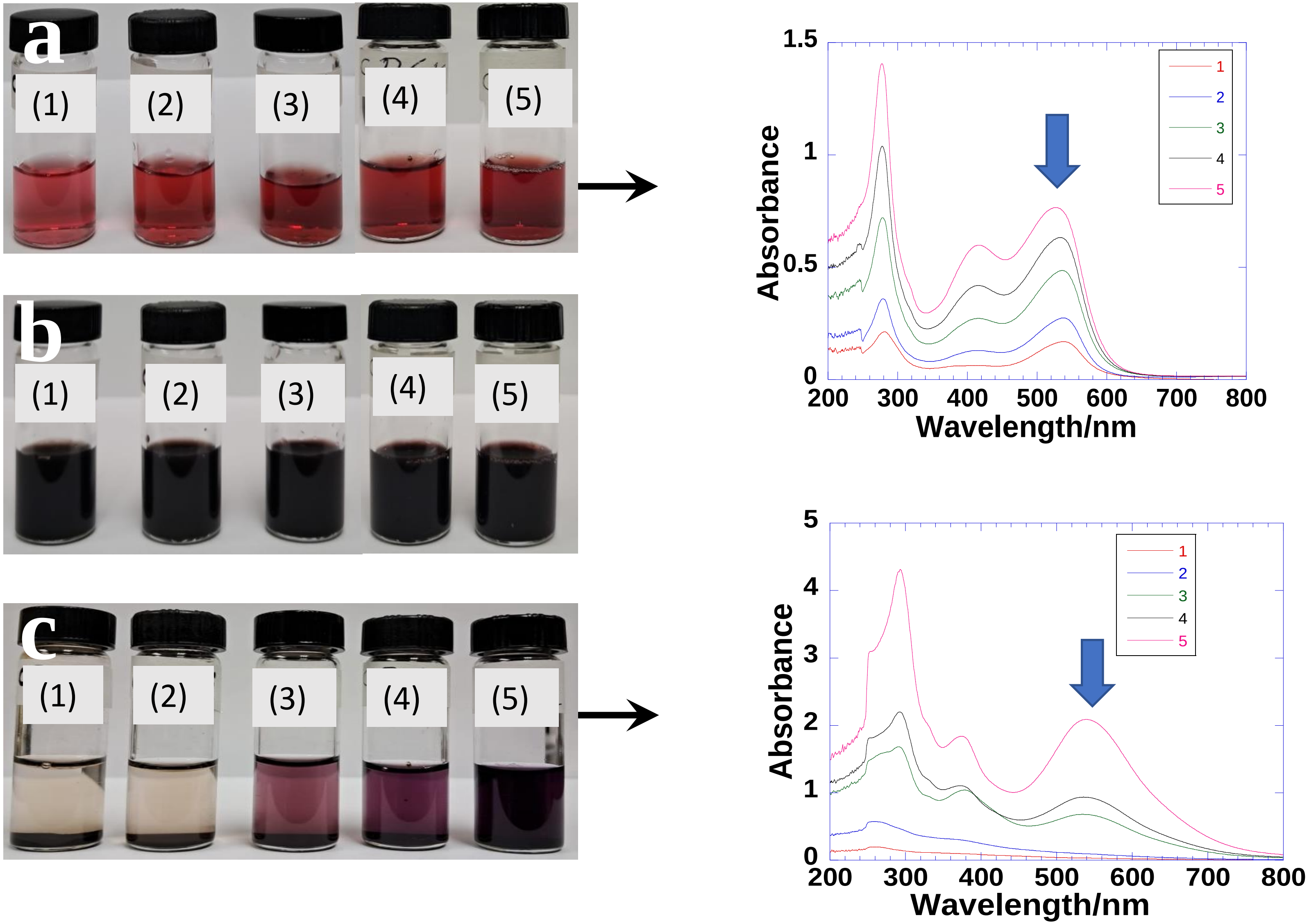

Fig S7b. (a) Photos of aqueous Pyrogallol Red solutions of (1) 0.05, (2) 0.1, (3) 0.2, (4) 0.4, and (5) 0.8 mM. (b) Photos after the addition of 5mM β-Cyclodextrin functionalized magnetic NPs in each bottle. (c) Photo after one week. Corresponding UV-visible spectra of the solution of each sample bottle of (a) and (c).

Fig S7c

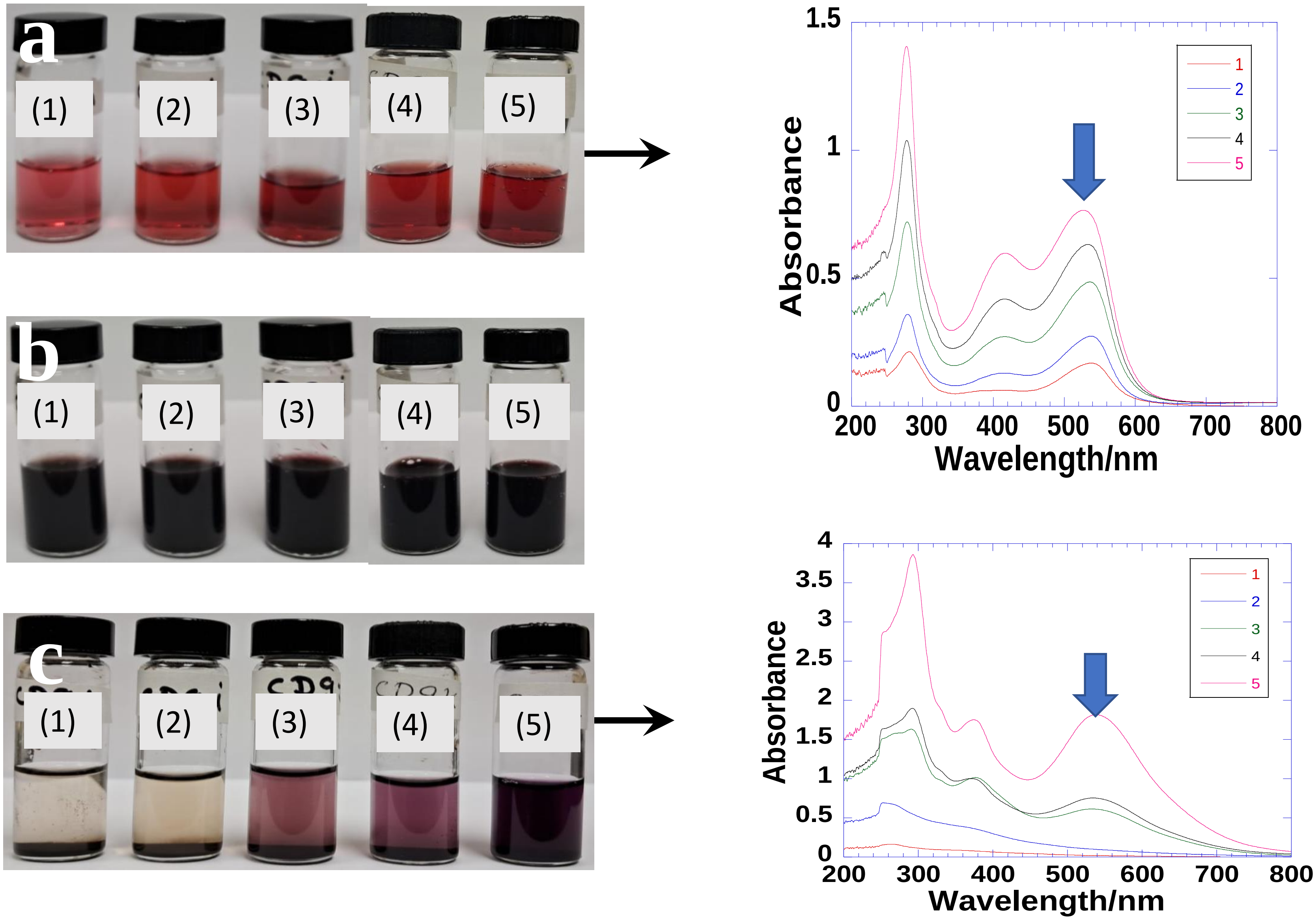

Fig S7c. (a) Photos of aqueous Pyrogallol Red solutions of (1) 0.05, (2) 0.1, (3) 0.2, (4) 0.4, and (5) 0.8 mM. (b) Photos after the addition of 5mM γ-Cyclodextrin functionalized magnetic NPs in each bottle. (c) Photo after one week. Corresponding UV-visible spectra of the solution of each sample bottle of (a) and (c).

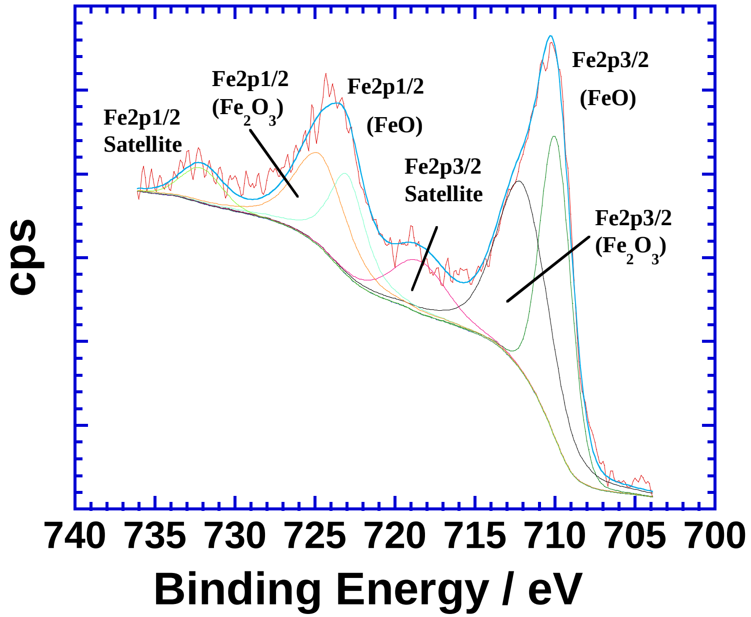

Fig S8. High resolution XPS spectra of Fe-2p of Red 40 dye loaded 16-6-16 functionalized magnetic NPs.

Fig S9

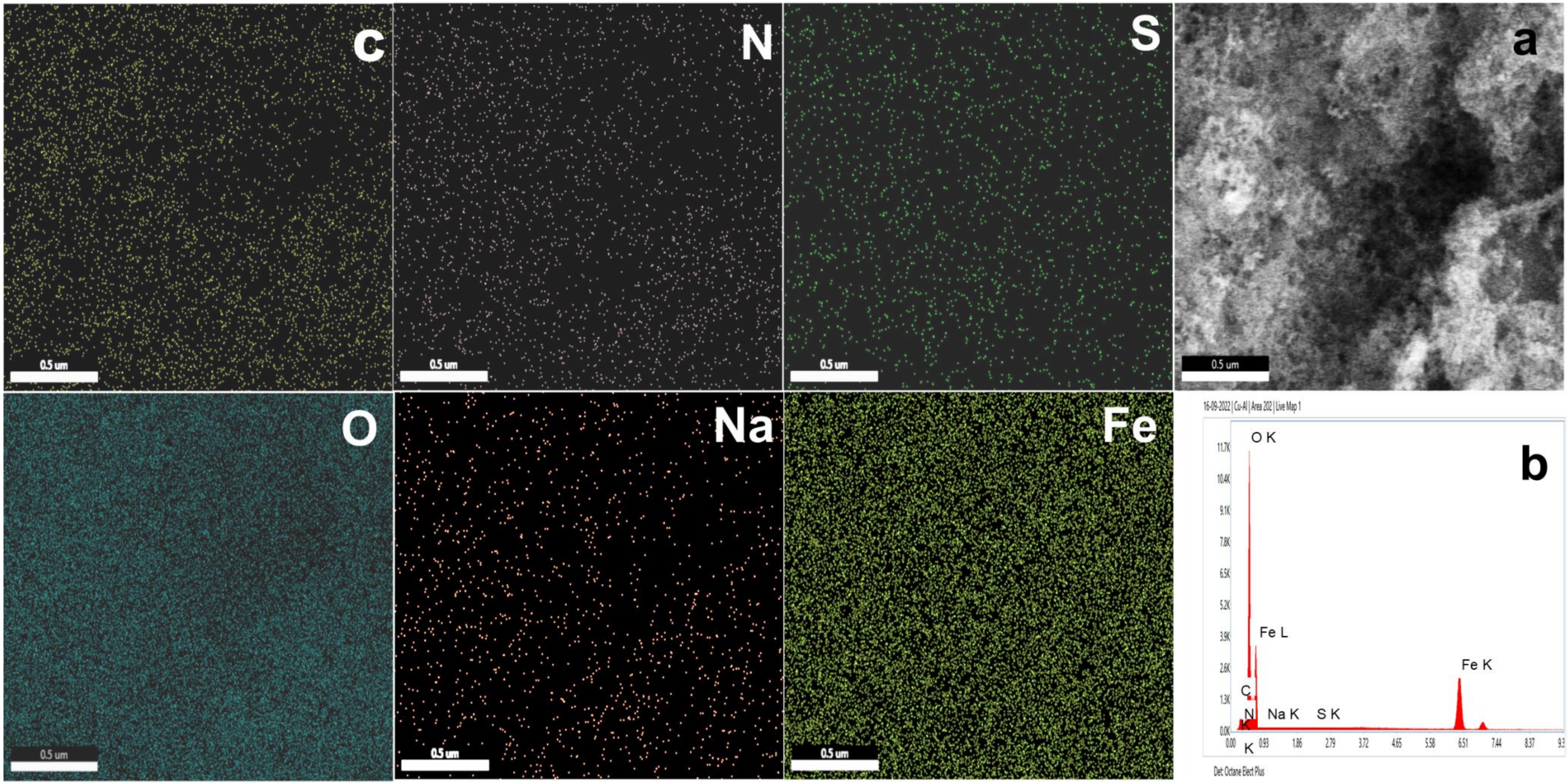

Fig S9. (a) FESEM image of Yellow 6 loaded 16-6-16 functionalized magnetic NPs. (b) EDS spectrum of different elements. Frames C, N, S, O, Na, and Fe represent the elemental mapping.

Fig S10

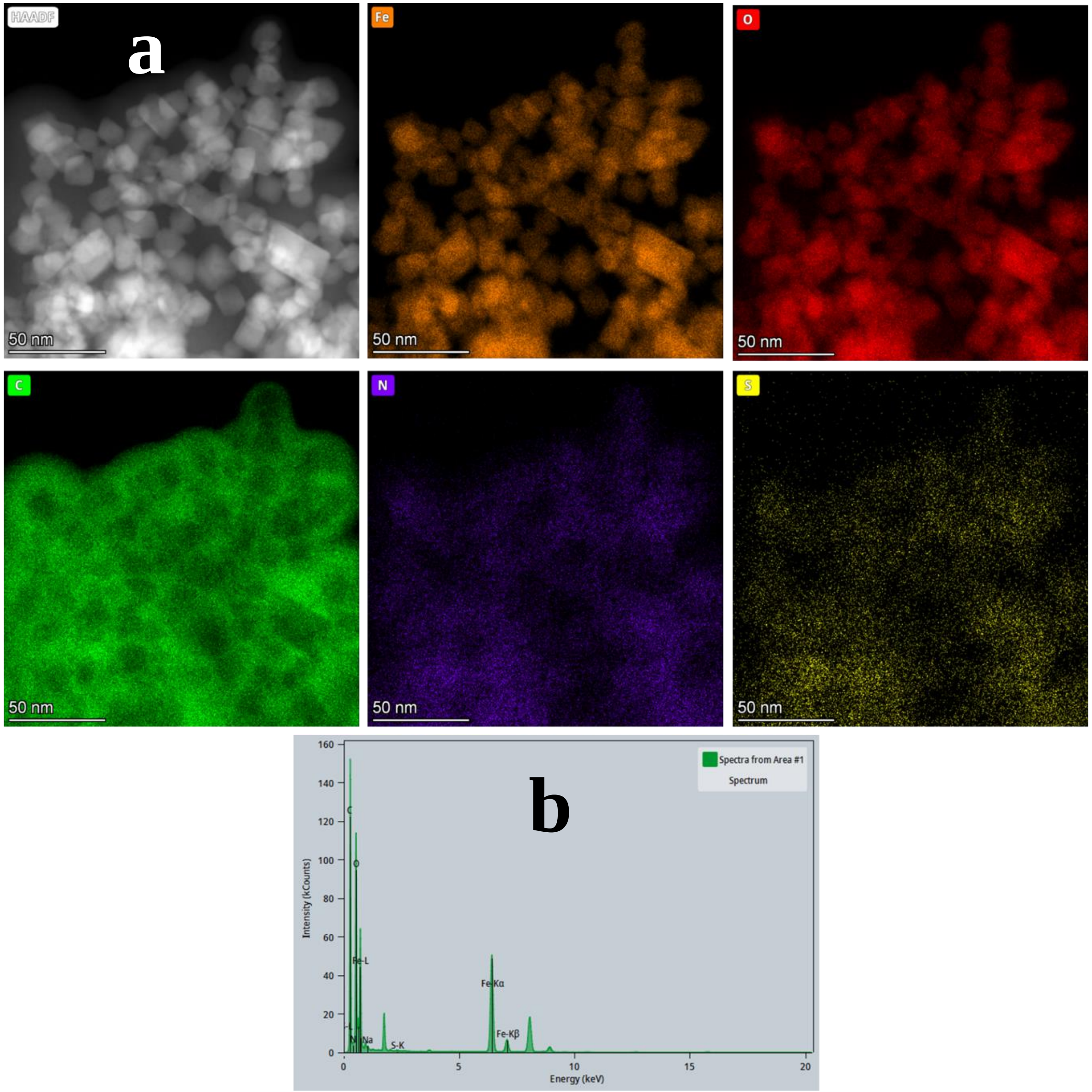

Fig S10. (a) High-angle annular dark-field (HAADF) TEM images of Red 40 loaded 16-6-16 functionalized iron oxide NPs. Frames Fe, O, C, N, S, and Na represent the elemental mapping. (b) EDS spectrum of various elements.

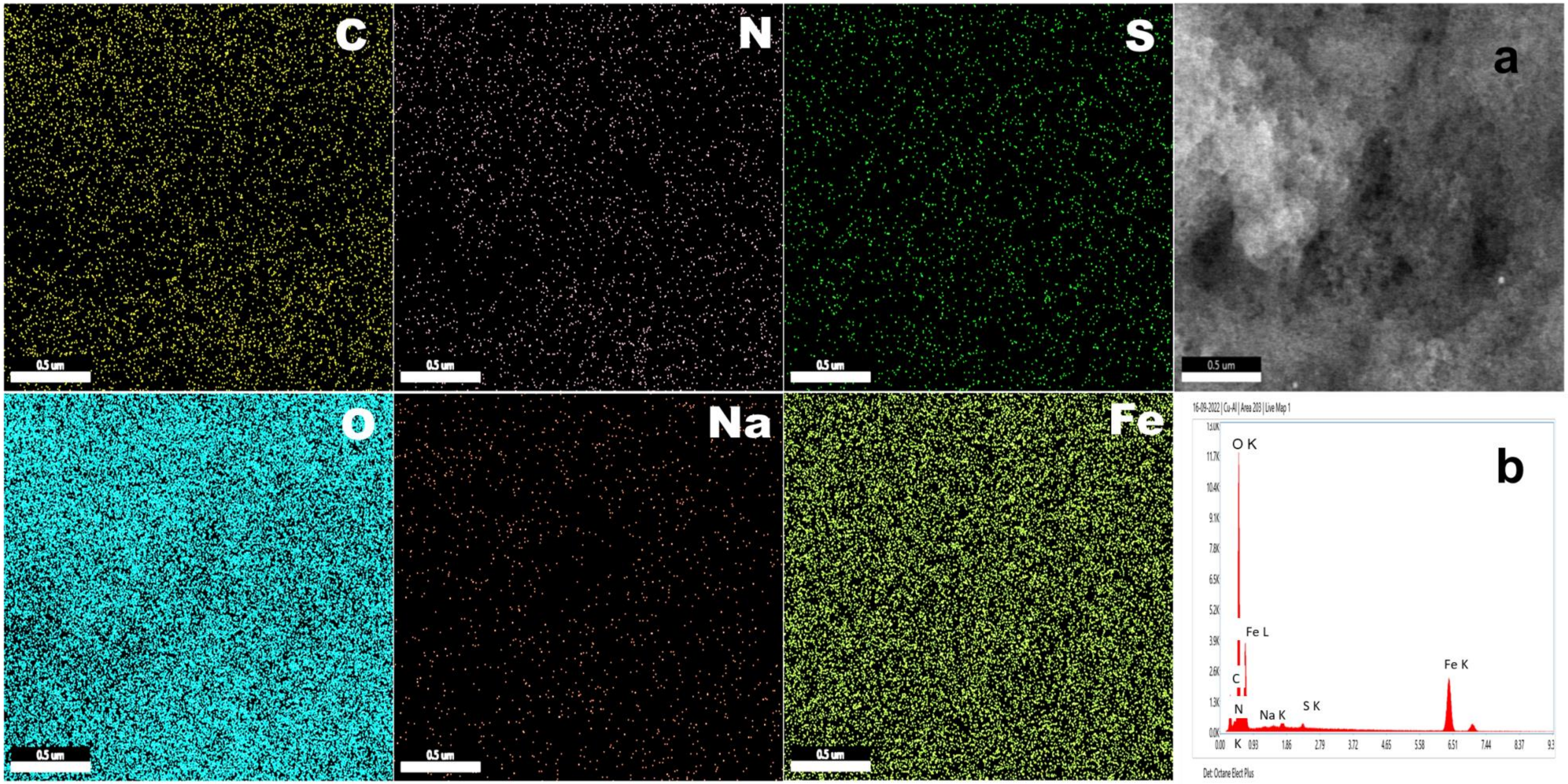

Fig S11. (a) FESEM image of Black T loaded b-D functionalized magnetic NPs. (b) EDS spectrum of different elements. Frames C, N, S, O, Na, and Fe represent the elemental mapping.

Table S1. “maximum concentration” / mM of dye extracted by 40 mM functionalized magnetic NPs at room temperature within a time period of one week evaluated by using UV-visible studies.

Functionalized magnetic NPs	dyes							
	Red 40	Yellow 5	Yellow 6	Blue 1	CBB	P Red	C Red	Black T
CTAB	0.05	0.05	0.1	0.05	0.1	0.35	0.05	0.35
16-6-16	0.1	0.1	0.1	0.05	0.35	0.7	0.1	0.35
TriCAT	0.2	0.2	0.2	0.2	0.4	0.8	0.3	0.4
HPS	<0.05	<0.05	<0.05	<0.05	<0.05	0.1	<0.05	0.05
α-CD pH = 7					0.1	0.1	0.1	0.1
β-CD pH = 7					0.1	0.1	0.1	0.1
γ-CD pH = 7					0.1	0.1	0.1	0.1
β-CD pH = 12					0	0.4	0.05	0.8
β-CD pH = 2					0.8	0.4	0	0.8

*Uncertainties in the measurements is ± 10 %.

Table S2a. IR frequencies of pure components, without and with dye loaded magnetic NPs. IR of all samples are performed in the dried state.

Blank MNPs (cm^{-1})	Surfactant functionalized magnetic NPs (cm^{-1})	Pure CTAB (cm^{-1})	Pure TriCAT (cm^{-1})	Pure Yellow 6 (cm^{-1})	Yellow 6 loaded TriCAT functionalized magnetic NPs (cm^{-1})	Yellow 6 loaded CTAB functionalized magnetic NPs (cm^{-1})
3447.55 $(-OH)_{str}$		3016 $(N-CH_3)_{as}$	3393 (N-H,1° amine)$_{str}$ 3008 $(N-CH_3)_{as}$,	3431 (N-H,1° amine)$_{str}$		3420 (N-H,1° amine)$_{str}$
		2943 $(CH_3)_{as}$, 2870 $(CH_3)_{str}$	2953 $(CH_3)_{as}$, 2919 $(CH_2)_{as}$		2919 $(CH_2)_{as}$ 2850 $(CH_2)_{str}$	2924 $(CH_3)_{as}$ 2829 $(CH_2)_{str}$
		2917$(CH_2)_{as}$, 2849 $(CH_2)_{str}$	2849 $(CH_2)_s$			
	2112 $(CO_2)_{str}$		2512,2467 (overtone)			
1653, 1635 $(-OH)_{bending}$				1619, 1550,1503 $(-N=N-)_{str}$ & $(-N-H)_{bending}$, $(C-C\ ring)_{str}$	1653 $(-N-H)_{bending}$	1652,1558,1503 $(-N=N-)_{str}$ & $(-N-H)_{bending}$, $(C-C\ ring)_{str}$
		1468 $(-CH_2)_{scissoring}$	1482,1471,1465 $(-CH_2)_{scissoring}$		1457 $(-CH_2)_{scissoring}$	1488 $(-CH_2)_{scissoring}$
	1394 $(N-CH_3)_{str}$	1394$(N-CH_3)_{str}$, 1360 $(CH_2)_{wagging}$	1376 $(C-H)_{bending}$	1390 $(C-H)_{bending}$, $(N-CH_3)_{str}$	1376 $(C-H)_{bending}$	
				1176, 1117 $(-SO3-)_{str}$		
			1009 $(-C-N)_{str}$	1030,1005 $(-C-N)_{str}$	1041 $(-C-N)_{str}$	1031 $(-C-N)_{str}$
		911 $(-C-N)_{str}$	984,953 $(C=C)_{bending}$	984 $(C=C)_{bending}$		
				898,831,800 $(-N-H)_{wagging}$	842 $(-N-H)_{wagging}$	
630.58 $(Fe-O)_{str}$	601,583,561 $(Fe-O)_{str}$, $(C-H)_{bending}$	720 $(CH_2)_{rocking}$	731,718 $(C-H)_{out\ of\ plane\ bending}$	745,707,668,634,597 (aromatic C-H)$_{out\ of\ plane\ bending}$	620,598,563 $(Fe-O)_{str}$ $(C-H)_{bending}$	587,556 $(Fe-O)_{str}$ $(C-H)_{bending}$

Table S2b. IR frequencies of pure components, without and with dye loaded magnetic NPs. IR of all samples are performed in the dried state.

**Blank MNPs (cm^{-1})**	**Surfactant functionalized magnetic NPs (cm^{-1})**	**Pure TriCAT (cm^{-1})**	**Pure P Red (cm^{-1})**	**P Red loaded TriCAT functionalized magnetic NPs (cm^{-1})**
3447<br>(-OH)$_{str}$		3393, (N-H,1° amine)$_{str}$<br>3008 (N-CH_3)$_{as}$,	3177<br>(O-H)$_{str}$	3374<br>(O-H,N-H-1° amine)$_{str}$
		2953 (CH_3)$_{as}$,<br>2919 (CH_2)$_{as}$		2923<br>(CH_3)$_{as}$
		2849<br>(CH_2)$_{str}$		2852<br>(CH_2)$_{str}$
	2112<br>(-CO_2)$_{str}$	2512, 2467<br>(overtone)		2129<br>(-CO_2)$_{str}$
1653,1635<br>(-OH)$_{bending}$			1614, 1575,1540,1506<br>(-C-C ring)$_{str}$	1600,1566,1504<br>(-N=N-)$_{str}$ &<br>(N-H)$_{bending}$,(C-C ring)$_{str}$
		1482,1471,1461<br>(-CH_2)$_{scissoring}$	1471, 1436, 1410<br>(O-H plane)$_{bending}$	1444, 1403<br>(O-H) $_{plane\ bending,}$<br>(-CH_2)$_{scissoring}$
	1394<br>(N-CH_3)$_{str}$	1376<br>(C-H)$_{bending}$	1369, 1330, 1302<br>(C-H)$_{bending}$, (C-O)$_{str}$	1334<br>(C-O)$_{str}$
			1127, 1104<br>(-C-H plane)$_{bending}$	1196, 1138<br>(-C-H plane)$_{bending}$
		1009<br>(-C-N-)$_{str.}$	1038,1010<br>(Ring deformation)$_{in\text{-}plane\ bending}$	1045 (-C-N-)$_{str,}$<br>(Ring deformation)$_{in\text{-}plane\ bending}$
		984,953<br>(C=C)$_{bending}$	973<br>(C=C)$_{out\text{-}of\text{-}plane\ bending}$	
			854,820<br>(O-H)$_{out\text{-}of\text{-}plane\ bending}$	883<br>(O-H)$_{out\text{-}of\text{-}plane\ bending}$
630<br>(Fe–O bond)$_{str}$,	601, 583,561<br>(Fe –O)$_{str}$,<br>(C-H)$_{bending}$	731,718<br>(C-H)$_{out\ of\ plane\ bending}$	657,634,613,553<br>(aromatic C-H)$_{out\ of\ plane\ bending}$	789, 736, 614, 588<br>(Fe –O bond)$_{str}$,<br>(C-H)$_{bending}$
